# Effects of the Pelotas (Brazil) Peace Pact on violence and crime: a synthetic control analysis

**DOI:** 10.1016/j.lana.2023.100447

**Published:** 2023-02-21

**Authors:** Michelle Degli Esposti, Carolina V.N. Coll, Eduardo Viegas da Silva, Doriam Borges, Emiliano Rojido, Alisson Gomes dos Santos, Ignacio Cano, Joseph Murray

**Affiliations:** a Human Development and Violence Research Centre, Federal University of Pelotas, Pelotas, Brazil; b Postgraduate Program in Epidemiology, Department of Social Medicine, Federal University of Pelotas, Pelotas, RS, Brazil; c Laboratório de Análise da Violência, Instituto de Ciências Sociais, Universidade do Estado do Rio de Janeiro, Rio de Janeiro, Brazil; d Instituto de Pesquisa Econômica Aplicada (IPEA), Brazil; e Instituto de Investigaciones Sociales, Universidad Autónoma de México, City of Mexico, Mexico

**Keywords:** Violence prevention, Public health, Synthetic control methodology, SCM, Synthetic control methodology, ITS, Interrupted time series, UN, United Nations, Pacto, Pelotas Pact for Peace

## Abstract

**Background:**

City-led interventions are increasingly advocated to achieve the UN's Sustainable Development Goal to reduce violence for all. We used a new quantitative evaluation method to examine whether a flagship programme, called the “Pelotas Pact for Peace” (the Pacto), has been effective in reducing violence and crime in the city of Pelotas, Brazil.

**Methods:**

We used synthetic control methodology to assess the effects of the Pacto from August 2017 to December 2021, and separately before and during the COVID-19 pandemic. Outcomes included monthly rates of homicide and property crime, and yearly rates of assault against women and school drop-out. We constructed synthetic controls (counterfactuals) based on weighted averages from a donor pool of municipalities in Rio Grande do Sul. Weights were identified using pre-intervention outcome trends and confounders (sociodemographics, economics, education, health and development, and drug trafficking).

**Findings:**

The Pacto led to an overall 9% reduction in homicide and 7% reduction in robbery in Pelotas. These effects were not uniform across the full post-intervention period as clear effects were only seen during the pandemic period. A 38% reduction in homicide was also specifically associated with the criminal justice strategy of Focussed Deterrence. No significant effects were found for non-violent property crimes, violence against women, and school dropout, irrespective of the post-intervention period.

**Interpretation:**

City-level interventions that combine public health and criminal justice approaches could be effective in tackling violence in Brazil. Continued monitoring and evaluation efforts are increasingly needed as cities are proposed as key opportunities for reducing violence for all.

**Funding:**

This research was funded by the 10.13039/100010269Wellcome Trust [grant number: 210735_Z_18_Z].

**Research in context**

**Evidence before this study**

It is unclear whether violence prevention programmes implemented by cities and municipalities are effective. We searched for evaluations of municipal-level or city-led interventions aimed at preventing crime and/or violence. We searched EMBASE, MEDLINE, Global Health, Pubmed, and PsycINFO up to January 25, 2022 (with no specified earliest date), with the search terms capturing the type of intervention ((municipal∗ AND (intervention OR program∗)) OR “public health intervention” OR “public health approach” OR “program evaluation” OR “community intervention” OR (city∗ ADJ intervention)) and outcomes (crime OR violen∗ OR homicide) of interest for this study. We also contacted key field experts for relevant articles. The search returned 1721 articles, of which 13 quantitative evaluations (11 empirical and two reviews) of violence and/or crime prevention programmes delivered at the municipality level or equivalent. Five studies were conducted in the United States, examining the impacts of Cure Violence and Focussed Deterrence programmes, and two studies were conducted in Brazil, examining post-1999 public policies in São Paulo and a violence prevention programme in Belo Horizonte, Minas Gerais. We also identified a systematic review on “what works” in reducing homicide in Brazil. This review highlighted a lack of robust evaluation evidence and called for rigorous examinations of violence prevention programmes at the municipal level.

**Added value of this study**

This is the first synthetic control analysis of the impacts of the city-led flagship violence prevention programme Pelotas Pact for Peace (the “Pacto”) in Brazil. Using a new quasi-experimental method, alongside stakeholder interviews, we identified that a municipal-led intervention led to reductions in serious violence. Stakeholders isolated the multi-sector infrastructure component of the intervention to be critical for not only tackling violence, but for also preparing cities to better respond to unplanned challenges, such as the COVID-19 pandemic.

**Implications of all the available evidence**

Our evaluation evidence provides initial support for city-led violence prevention programmes as effective tools for tackling violence. There is an urgent need for ongoing monitoring and robust evaluation of programmes being introduced in cities across Brazil, and worldwide, to identify what works and what does not work in preventing violence for all.

## Introduction

Violence and crime have been steadily rising in Brazil since the 1980s, usurping the death toll caused by infectious diseases.^1^, ^2^, ^3^ Violence is now the leading cause of death among people aged 15–49 years old, and in 2019 more than 65,900 people lost their lives to interpersonal violence—representing the highest number of violent deaths globally.^2^ Brazil thus faces, and presents, a major challenge in achieving the United Nation's Sustainable Development Goal (SDG) 16.1 to reduce violence for all by 2030.^4^

Growing evidence points to public health and criminal justice approaches to achieve violence reduction.^5^^,^^6^ Public health approaches, such as Cure Violence, benefit from the inclusion of stakeholders, community buy-in, and addressing multiple causes of violence in primary prevention.^7^, ^8^, ^9^ Criminal justice approaches, such as hot spots policing and Focussed Deterrence, benefit from the effective use of data and resources to manage high-risk areas and individuals.^10^, ^11^, ^12^ Research has called for a better link between public health and criminal justice approaches,^13^, ^14^, ^15^ and policy has advocated for cities as vehicles for delivering interventions.^16^^,^^17^ Local governments across Brazil have begun to action these calls, developing integrated violence prevention programmes to tackle violence and crime in their municipalities. However, a recent systematic review of what works in reducing homicide in Brazil identified a lack of robust evaluation evidence of municipal-level interventions, with only good evidence of effectiveness for one violence prevention programme in Belo Horizonte, Minas Gerais.^18^^,^^19^

Since 2000, the city of Pelotas in Rio Grande do Sul saw an unprecedented surge in violence and crime, which exceeded average municipal-level increases across Brazil. Homicide rates increased by five-fold from 6.6 per 100,000 residents in 2000 to 31.5 in 2017.^1^ In response, the Pelotas Municipal Government designed and delivered an innovative multi-sector programme that aimed to prevent violence and reduce crime across the city: the Pelotas Pact for Peace, the “Pacto”.^20^ The programme incorporated elements from public health and criminal justice approaches, including targeting early risk factors (e.g., school-based interventions) and engaging gang leaders to address homicide (e.g., Focussed Deterrence). The Pacto has since gained international recognition as a flagship city-led violence prevention programme, and Pelotas has been named as the third Pathfinder City by the United Nation's Secretary-General Global Partnership.^21^

Despite this, the impacts of the Pacto are unclear, leaving a critical evidence gap. Public reports and events have presented the programme as a vast success,^22^^,^^23^ while the only external evaluation was unable to identify a clear impact of the Pacto—as a whole—since its launch in August 2017 up until September 2019.^24^ A narrower examination of the Pacto's Focussed Deterrence component identified around 65 fewer homicides during its implementation from May 2018, but it was unclear whether this reduction can be attributed to the intervention itself due to confounding from regression to the mean following a spike in homicide caused by a spree of revenge gang killings shortly before the Focussed Deterrence component was introduced.^20^^,^^24^ Additional evidence is needed to determine the effectiveness of the Pacto and, given that many interventions were implemented with a view to long-term effects, there is further need for continuing evaluation. This is critical for informing the intervention going forward and advancing understanding on what works for preventing violence, particularly in high violence regions.

We use a new quasi-experimental method to continue to monitor the effects of the Pacto. Synthetic control methodolody (SCM) overcomes previous challenges of effect identification and offers an evaluation approach which can flexibly handle the gradual introduction of a complex intervention at the municipal-level.^25^ Informed by qualitative insights from stakeholder interviews, we used SCM to estimate the causal effects of the Pacto on violence and crime before and during the COVID-19 pandemic.

## Methods

Routinely collected data from police, health, and education records were used to examine monthly and yearly rates of violence and crime in Pelotas, from January 2012 to December 2021. We used SCM, which extends a controlled before-after study design to assess the effects of an intervention. In this study, SCM estimated what would have happened if the Pacto had not taken place in Pelotas. We pre-registered our analysis plan at the Open Science Framework (osf.io/c5v2a) and obtained ethical approval from the Research Ethics Committee of the Federal University of Pelotas School of Medicine (CAAE registration number: 53190721.0.0000.5317).

### Pelotas Peace Pact

The Pelotas Municipal Government, in partnership with *Comunitas* and the *Instituto de Cidade Segura* (Safe City Institute), developed a new municipal violence prevention policy that launched in August 2017: *Pacto Pelotas pela Paz* (“Pacto”), or Pelotas Peace Pact in English.^20^^,^^24^ The Pacto aimed to reduce violence and crime by combining public health and criminal justice approaches through a series of primary, secondary, and tertiary prevention strategies. The Pacto is a complex multi-sector initiative that is structured in five axes: Social Prevention, Policing and Justice, Administration, Urbanism and Technology (web-appendix p 7). While all axes aimed to reduce violence—including early risk factors of violence (e.g., school dropout)—the Policing and Justice Axis most directly targeted violence and crime and included the criminal justice strategy of Focussed Deterrence, implemented from May 2018. This strategy specifically targeted homicide by directly communicating the consequences of engaging in lethal violence to imprisoned gang leaders.^24^ A proposed logic model of the impact of the Pacto on violence and crime is shown in web-appendix (p 28).

### Stakeholder interviews

We conducted semi-structured qualitative interviews with key personnel in the Pacto to inform our logic models and establish whether strategic actions were implemented and adhered to both before and during the COVID-19 pandemic. A primary interview with the Pacto's Executive Secretariat was conducted to understand its overall structure, confirming the scope and implementation status of the different projects and obtaining information about any changes due to the pandemic. This guided additional interviews with professionals charged with delivering different strands of the Pacto, including key actors from the judiciary, police, prison system, as well as coordinators of social prevention projects. More details of the interviews are provided in the web-appendix (pp 3–5 and 8).

### Outcomes

Outcomes are summarised in web-appendix, pp 13 and 14. The main outcomes were monthly rates of police-recorded crime by municipality, Rio Grande do Sul, from January 2012 to December 2021. We extracted data on the number of victims of homicide and occurrences of property crime (thefts, robberies, vehicle thefts and robberies) and assault against women from the Secretariat for Public Security.^26^ We calculated rates per 100,000 person-years residing in each municipality using annual municipal population estimates based on the 2010 census.^27^ We also obtained school dropout (a targeted early risk factor, see web-appendix p 29) for elementary and middle schools from 2014 to 2021 via a Freedom of Information request to the State Secretary for Education.^28^ Dropout rates were calculated per 100,000 students initially enrolled in schools. Data on assault against women and school dropout were only available yearly (not monthly).

### Control variables

As well as matching on yearly pre-intervention trends in the study outcomes, we included confounders when constructing the synthetic controls (web-appendix, p 15). These included pre-intervention measures of population sociodemographics (age, sex, and race distributions), economics (% living in poverty, measures relating to the Bolsa Familia national cash transfer programme), education (% with higher education), human development index, and drug trafficking. In sensitivity analyses of the effects of the Pacto after March 2020, we also included measures relating to COVID-19 (related deaths and workplace mobility).

### Statistical analysis

SCM compares changes in outcomes in Pelotas before and after the Pacto with a “synthetic control” that is a weighted average from a pool of potential control units, the “donor pool”. The donor pool included 22 other municipalities in Rio Grande do Sul state with a similar population size to Pelotas (≥80,000 residents) but without similar city-wide interventions during the study period (Canoas and Lajeado were excluded^29^^,^^30^; web-appendix, p 32). The control weights are identified so that the synthetic control most closely resembles Pelotas before the Pacto was introduced. SCM uses data-driven algorithms to minimise the distance between a vector of characteristics (outcomes and confounders) for the treated and the synthetic control unit before the intervention. Any differences between the treated and the synthetic control unit after implementation can therefore be attributed to the intervention itself, provided that a good synthetic control fit is identified (web-appendix p 6 for advantages).^25^ Additional technical details for SCM is found in web-appendix p 6.

Synthetic control models were fitted to estimate the effect of the Pacto for the full 52 months post-intervention (since August 1, 2017), and before and during the COVID-19 pandemic (starting from March 1, 2020; see web-appendix, p 16). We also fitted synthetic control models to estimate the effect of the Focussed Deterrence strategy on homicide, starting in May 1, 2018. In line with previous recommendations, we used the synthetic control to conduct a controlled interrupted time series (ITS) analysis and test for difference-in-differences in the level and trend change in Pelotas compared to synthetic control (a three-way interaction).^31^ A level change would imply that the Pacto had an immediate effect, while a trend change would mean that it gradually impacts the outcome over time (e.g., slowing or reversing the previously increasing trend in homicide). We specified Newey–West standard errors to account for heteroskedascity and autocorrelation, as well as uncertainty from estimating the synthetic control.^32^ This methodological extension allows for statistical inference and was prioritised over placebo tests and robust t-tests (web-appendix p 6) due to its ability to simultaneously assess and account for any possible imperfect synthetic control fit via testing for pre-intervention differences between Pelotas and the synthetic control.

We performed various sensitivity analyses to check the robustness of our findings. This included replicating analyses across data sources (police vs health-recorded homicide, web-appendix p 6), controlling for COVID-19 measures, pre-filtering (smoothing) the outcome series to overcome noisy outcome data issues and possible overfitting,^33^ and excluding Porto Alegre (the State's Capital) from our donor pool.^34^ All analyses were conducted in R (version 4.10) using the *Synth* package.

### Role of funding source

The funder of the study had no role in the study design, data collection, data analysis, data interpretation, or writing of the report. All authors had full access to the data and had the final responsibility for the decision to submit for publication.

## Results

There were 704 homicides, 28,160 robberies, 38,665 thefts, and 6839 vehicle robberies and thefts in Pelotas from January 1, 2012, through December 31, 2021. Although monthly violence and crime rates were lower following the introduction of the Pacto in August 2017, lower rates were also seen across other municipalities during this period (web-appendix, pp 17 and 18).

### Homicide

**Fig. 1:**
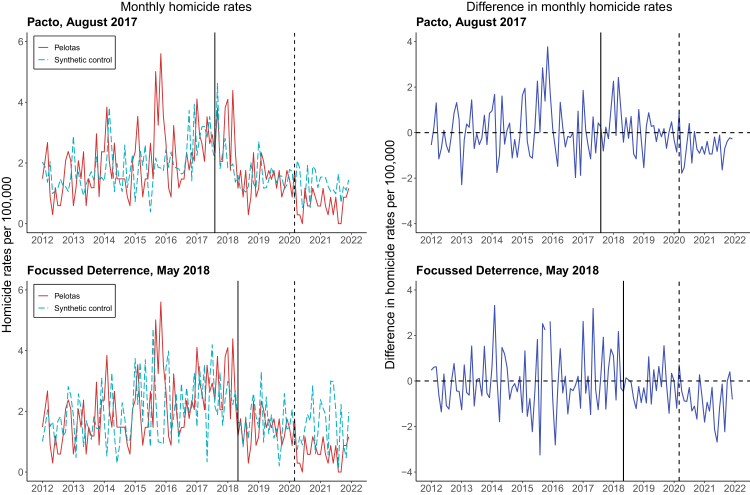
**Monthly police-recorded homicide rates (per 100,000 residents) before and after the introduction of the Pelotas Peace Pact and Focussed Deterrence, compared with synthetic controls, before and during the COVID-19 pandemic.** The solid vertical line represents the introduction of the intervention of Pelotas Peace Pact in August 2017 (*top panels*) and Focussed Deterrence in May 2018 (*bottom panels*); and the dashed vertical line represents the beginning of the COVID-19 period in South Brazil. The solid red lines represent the treated municipality of Pelotas and the dashed light blue lines represent the synthetic control *(left panels)*, the solid dark blue lines represent the difference between Pelotas and the synthetic control *(right panels)*.

Compared to its synthetic control (web-appendix, pp 19–21), Pelotas had 9% fewer monthly homicides (Table 1 & Fig. 1)—equivalent to 5 fewer homicides each year or 23 fewer homicides over the full 52 months after implementation. This overall effect included an initial period of 13% more homicides in Pelotas before the COVID-19 pandemic, followed by 47% fewer homicides during the pandemic. Following the Focussed Deterrence strategy in May 2018, there were 38% fewer homicides in Pelotas compared to its synthetic control, with a more pronounced 55% reduction during the pandemic period. Findings were similar for health-recorded homicides, which had slightly attenuated estimates due to the shorter available time frame (web-appendix, p 22).

**Table 1:** Effects of Pelotas Peace Pact on violence, crime, and school dropout, before and during the COVID-19 pandemic, compared with synthetic control.

	Full post-intervention period			Before the COVID-19 pandemic			During the COVID-19 pandemic		
	Synthetic control	Pelotas	Difference, absolute (relative)	Synthetic control	Pelotas	Difference, absolute (relative)	Synthetic control	Pelotas	Difference, absolute (relative)
**Monthly police-recorded crime rates per 100,000 residents**									
**Pelotas Peace Pact, August 2017**									
Homicide	1.50	1.37	−0.13 (−9%)	1.65	1.86	0.21 (13%)	1.28	0.67	−0.61 (−47%)
Robbery	59.88	55.71	−4.16 (−7%)	73.28	74.63	1.35 (2%)	40.99	29.06	−11.93 (−29%)
Theft	78.36	76.87	−1.49 (−2%)	84.44	88.11	3.66 (4%)	69.78	61.04	−8.75 (−13%)
Vehicle theft & robbery	13.06	11.53	−1.53 (−12%)	15.97	15.3	−0.67 (−4%)	8.95	6.22	−2.74 (−31%)
**Focussed deterrence, May 2018**									
Homicide	1.67	1.04	−0.63 (−38%)	1.85	1.41	−0.45 (−24%)	1.5	0.67	−0.82 (−55%)
**Yearly rates of assault against women and school drop-out per 100,000**^a^									
**Pelotas Peace Pact, August 2017**									
Assault against women	417.78	401.32	−16.46 (−4%)	–	–	–	–	–	–
School dropout	2519.92	1439.93	−1079.99 (−43%)	–	–	–	–	–	–

Monthly crime rates span from January 1, 2012, until December 31, 2021, while yearly rates of assaults against women span from 2012 until 2021 and school dropout from 2014 until 2021. Before the COVID-19 pandemic spans up until February 28, 2020, and during the pandemic spans from March 1, 2020, onwards. The relative difference between Pelotas and the synthetic control is represented by the total percentage difference while the absolute difference is represented by the average monthly difference.

a Rates are derived using yearly female population estimates and total pupils initially enrolled in schools by municipality.

Controlled ITS analyses identified no significant step change immediately following the Pacto. However, there was a significant change in trend (Table 2 and web-appendix, p 43): although both the synthetic control and Pelotas showed a significant downward trend in homicide after the Pacto, this decrease was more-than-twice as pronounced in Pelotas (*B* = −0.04; 95%CI:−0.06, −0.02; *p* < 0.001). The introduction of the Focussed Deterrence strategy was associated with both a larger step reduction in homicide (*B* = −0.88; 95%CI:−1.52, −0.24; *p* = 0.0069) and a steeper downward trend (*B* = −0.02; 95%CI:−0.04, −0.01; *p* = 0.0040). However, there were also significant pre-intervention differences, indicating that good synthetic control fit was not achieved for Focussed Deterrence (Table 2). Quasi *p* values derived from placebo tests and robust t-tests, however, did not identify any significant effects (web-appendix, pp 23,33–36).

**Table 2:** Controlled interrupted time series analyses estimating the significance of the effects of Pelotas Peace Pact on violence and crime, full post-intervention period.

	Effect estimates (*B*, 95% CI)					
	Pre-intervention		Post-intervention			
	Difference in level (Synthetic control—Pelotas)	Difference in trend (Synthetic control—Pelotas)	Change in level (pre—post intervention)	Difference in change in level (Synthetic control—Pelotas)	Change in trend (pre—post intervention)	Difference in change in trend^a^ (Synthetic control—Pelotas)
**Monthly rates per 100,000 residents**						
**Pelotas Peace Pact, August 2017**						
Homicide						
Synthetic control	−0.34 (−0.8, 0.11)	0.01 (0, 0.02)	−0.55 (−0.87, −0.22)∗∗∗	0.33 (−0.62, 1.28)	−0.03 (−0.03, −0.02)∗∗∗	−0.04 (−0.06, −0.02)∗∗∗
Pelotas			−0.22 (−1.09, 0.65)		−0.07 (−0.09, −0.05)∗∗∗	
Robbery						
Synthetic control	0.89 (−6.04, 7.83)	−0.01 (−0.18, 0.16)	−17.93 (−24.67, −11.18)∗∗∗	9.56 (−1.86, 20.97)	−1.81 (−2.02, −1.59)∗∗∗	−0.54 (−0.98, −0.09)∗
Pelotas			−8.37 (−17.57, 0.83)		−2.35 (−2.73, −1.96)∗∗∗	
Theft						
Synthetic control	2.71 (−14.4, 19.81)	−0.11 (−0.49, 0.26)	−10.71 (−22.1, 0.67)	13.11 (−3.28, 29.5)	0.09 (−0.52, 0.69)	−0.25 (−1.03, 0.52)
Pelotas			2.39 (−9.88, 14.67)		−0.17 (−0.61, 0.28)	
Vehicle theft & robbery						
Synthetic control	1.87 (−5.02, 8.76)	−0.05 (−0.22, 0.11)	−4.89 (−8.56, −1.23)∗∗	1.84 (−4.57, 8.25)	−0.33 (−0.42, −0.24)∗∗∗	−0.01 (−0.19, 0.18)
Pelotas			−3.05 (−8.33, 2.23)		−0.34 (−0.5, −0.18)∗∗∗	
**Focussed deterrence, May 2018**						
Homicide						
Synthetic control	−0.49 (−0.92, −0.07)∗	0.01 (0, 0.02)∗∗	−0.54 (−0.83, −0.24)∗∗∗	−0.88 (−1.52, −0.24)∗∗	−0.02 (−0.03, −0.02)∗∗∗	−0.02 (−0.04, −0.01)∗∗
Pelotas			−1.42 (−1.98, −0.86)∗∗∗		−0.05 (−0.06, −0.03)∗∗∗	

Effects estimated from controlled (or multiple-group) interrupted time series (ITS) analyses to test for difference-in-differences in crime trend in Pelotas, compared to synthetic control, before the COVID-19 pandemic and for the full intervention period.^31^

∗*p* < .05; ∗∗*p* < .01; ∗∗∗*p* < .001.

a Effect estimate represents the test of difference-in-differences in crime trend (i.e., a three-way interaction: trend x intervention time-point x treated unit).

### Property crime

**Fig. 2:**
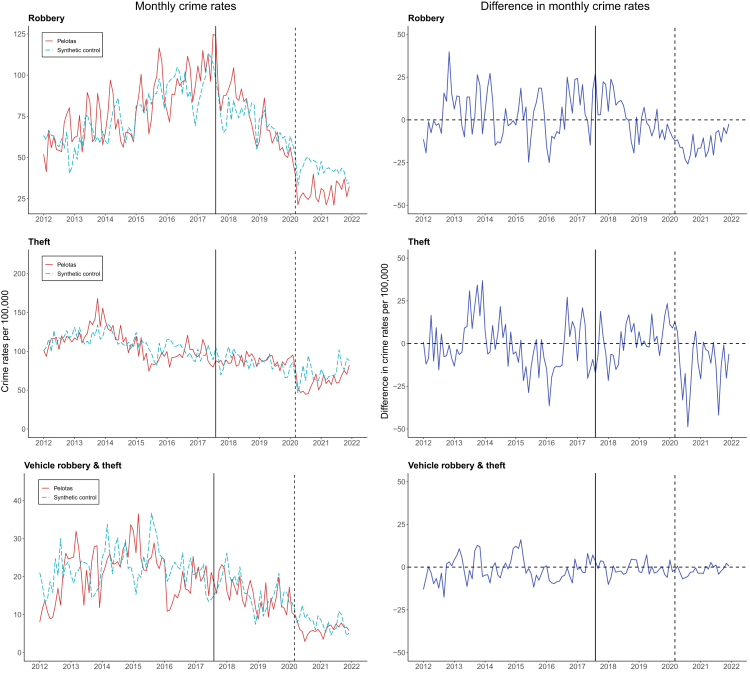
**Monthly property crime rates (per 100,000 residents) before and after the introduction of Pelotas Peace Pact, compared with synthetic controls, before and during the COVID-19 pandemic.** The solid vertical line represents the introduction of Pelotas Peace Pact and the dashed vertical line represents the beginning of the COVID-19 period in South Brazil. The solid red lines represent the treated municipality of Pelotas and the dashed light blue lines represent the synthetic control *(left panels)*, the solid dark blue lines represent the difference between Pelotas and the synthetic control *(right panels)*.

Crime rates were 7% lower for robbery, 2% lower for theft, and 12% lower for vehicle robbery and theft in Pelotas compared to the synthetic control (Table 1 & Fig. 2). These reductions translated to around 740 fewer robberies and 270 fewer thefts and vehicle thefts and robberies across the full post-intervention period. Similarly to homicides, these differences mainly referred to the COVID-19 period (range: 13%–31%). For example, robbery rates were 2% higher in Pelotas than the synthetic control before the pandemic, yet 29% lower during the pandemic period. Tests of difference-in-differences revealed a significant effect of the Pacto on robbery (Table 2 & web-appendix p 44), whereby Pelotas had a steeper decreasing trend following the Pacto than the synthetic control (*B* = −0.54; 95%CI:−0.98, −0.09; *p* = 0.017). The effects for theft and vehicle theft and robbery were consistently not significant (*p* > 0.05; Table 2 & web-appendix, pp 23, 37–42, 44).

### Yearly outcomes: assault against women and school dropout

**Fig. 3:**
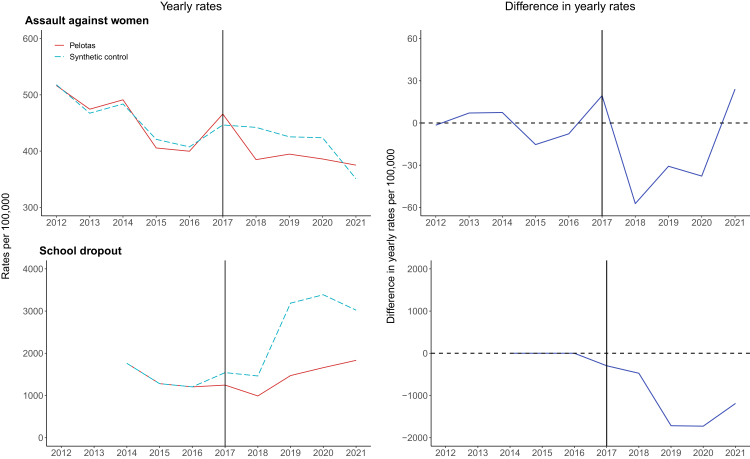
**Yearly rates of assault against women and school dropout before and after the introduction of the Pelotas Peace Pact, compared with synthetic controls.** The solid vertical line represents the introduction of Pelotas Peace Pact since these outcome data are yearly (not monthly). For assault against women rates are derived using yearly female population estimates and for school dropout rates are derived using the total of pupils initially enrolled in schools by municipality. The solid red lines represent the treated municipality of Pelotas and the dashed light blue lines represent the synthetic control *(left panels)*, the solid dark blue lines represent the difference between Pelotas and the synthetic control *(right panels)*.

Compared to the synthetic control (web-appendix, pp 19 & 24), there was a small 4% reduction in assault against women (Table 1 & Fig. 3). School dropout rates were also 43% lower in Pelotas since the synthetic control experienced marked increases in school dropout rate which did not occur to the same extent in Pelotas. However, placebo tests indicated that these effects were not significant (web-appendix, p 23) and there were too few pre-intervention time-points to conduct controlled ITS analyses and robust t-tests.

### Robustness checks

Findings were robust to various sensitive analyses. Controlling for monthly COVID-19 measures did not change estimated effects (web-appendix, p 25), while pre-filtering the monthly outcome data and removing Porto Alegre (the state's capital) from the donor pool estimated marginally larger effects for homicide and robbery during the pandemic period (web-appendix, pp 26 and 27).

## Discussion

The roll-out of a multi-sector violence and crime prevention programme at the municipal-level in South Brazil, the Pelotas Peace Pact (the Pacto), was associated with an overall 9% reduction in homicide and 7% reduction in robbery. This meant that 23 homicides and 740 robberies were prevented by the Pacto. However, programme effects were not uniformly distributed over time. Increases in these serious violent crimes were initially seen immediately after the Pacto was launched, which were then followed by pronounced decreases during the COVID-19 pandemic. Irrespective of the post-intervention period examined, we found limited evidence of the effectiveness of the Pacto on thefts, vehicle thefts and robberies, and assault against women. The reliability of the estimated 43% reduction on school dropout after the Pacto was unclear since analyses were limited by only yearly data availability.

While the Pacto is associated with potentially meaningful reductions in homicide and robbery in Pelotas, our findings contrast local celebrations of the vast success of the programme.^22^ Official local government and national websites claim that the Pacto has led to a 73%-82% reduction in homicide and a 70% reduction in robberies.^23^, ^35^ These estimates are substantially larger than those identified here because comparable municipalities in the state also saw reductions in crime. Much of the improvements in violence and crime since August 2017 may instead be attributed to state-wide reductions due to factors that are not unique to Pelotas or the Pacto. For example, the human development index, which captures health, education, and standard of living, has consistently improved while income inequality has narrowed in Rio Grande do Sul since 2017.^27^ Additionally, the state has rolled out some public security initiatives aimed to “combat crime” during the study period (see limitations below).^36^

Traditionally non-federal states have been the primary actor in public security, but more recently there has been a shift towards municipalities.^17^^,^^37^ In Brazil, a growing number of former police officers are running for local office, and mayors are beginning to propose public security policies.^37^ Internationally, city-leaders have also been placed at the heart of pioneering interventions to reduce urban violence (cf Peace in Our Cities), and the Pacto is one of these flagship programmes.^38^ As municipalities and cities take a more active role in public security, it is increasingly important to evaluate effects of initiatives at the local level. Despite this, we found few evaluations of municipal-level interventions in Brazil.^18^^,^^19^ Although still limited, there is a larger evidence-base in the US suggesting that a combined public health and criminal justice approach is most effective in reducing violence at the city-level.^9^^,^^12^^,^^15^ Our evaluation thus adds to a critical evidence gap about city-level interventions for violence prevention.

Our findings underline the importance of ongoing monitoring and evaluation. The only previous evaluation of the Pacto identified no overall effect on homicides, only an effect restricted to Focussed Deterrence. However, our continued monitoring found more recent effects, beyond September 2019.^24^ There are different possible explanations as to why the Pacto initially was estimated to have no effect on homicide. First, we used the more rigorous evaluation design of SCM, which addresses many of the limitations of the previous single-unit ITS evaluation.^24^ Second, Pelotas saw a sudden spike in homicides shortly following the Pacto's introduction and just before the introduction of the Focussed Deterrence component — due to a spree of revenge killings between rival gang members.^39^ This unexpected spike in violence is not understood to relate to the Pacto itself and could have obscured short-term effects of the programme, but, on the other hand, it might invoke regression to the mean which could have biased previous estimates for Focussed Deterrence upwards.^34^ Third, any effects may not be immediate but gradual. Public health approaches are hypothesised to have delayed effects, compared to criminal justice tertiary prevention approaches, since they target early risk factors and social norms.^40^ Although our interviews identified that the Policing and Justice Axis programmes were most strongly implemented, and these are more likely to have immediate effects. Our interviews suggest that these immediate reductions may be more related to the harmonisation of activities across the civil police, military police, prosecutors, and courts, particularly related to violent property crime prevention.

In addition, it is important to consider the estimated effects of the Pacto in relation to changes occurring because of the COVID-19 pandemic. Multiple interviewees stated that Pelotas more strongly enforced COVID regulations than other municipalities, thus restricting mobility and exposure. This, combined with increased police visibility and improved coordination between sectors introduced by the Pacto itself, may have played a role in reducing crime in the city. However, it is also possible that there were other factors unique to Pelotas, not just the Pacto, which meant that the city saw larger-than-expected reductions in crime during this period. For example, Pelotas hosts two major universities, which together account for around 22,000 students. The suspension of classes and shift to remote learning for over one year may have had a unique impact on violence and crime in Pelotas, which would not have been seen in non-university municipalities. Indeed, evidence from the US suggests that the economy of university towns was more detrimentally affected during the pandemic,^41^ and this may have led to fewer opportunities for property crime, drug trafficking, and violent crime.^42^

Limitations of this evaluation include various external shocks—unrelated to the Pacto—which may threaten the validity of our effect estimates. Rio Grande do Sul state rolled out a series of crime combatting initiatives during the study period. Since July 2019, the state has bolstered police resources in 18 municipalities, including Pelotas and 14 of our control municipalities.^36^ Due to the staggered nature of this state-wide intervention, it is not possible to disentangle its effect from the Pacto but it seems unlikely to explain our estimates given that the intervention was also experienced in control municipalities. COVID-19 also represents an external shock. The pandemic disrupted many of the Pacto's planned activities, especially in the Social Prevention Axis (e.g., the school-based intervention programme *Cada Jovem Conta*). This could explain why there were no consistent effects on school reduction throughout the full post-intervention period. Methodologically we were limited by data availability; with consistent monthly crime data only being available for the full study period on homicide and property crimes, and temporal delays in reporting and classifying crimes (especially theft; see web-appendix, p 14). These various external shocks, the disruption of the pandemic on programme adherence, and data restrictions, undermine casual inference. These obstacles reflect challenges in evaluating real-life interventions.

### Conclusions

City-level interventions are increasingly proposed as key opportunities for achieving the Sustainable Development Goal (SDG) 16.1 to reduce violence for all.^16^ Despite a growth in municipal-level interventions across Brazil, and city-led interventions around the world, it is still unclear how effective these approaches are in tacking violence. In this study, we conducted a rigorous quasi-experimental evaluation of an internationally recognised violence prevention programme in Brazil to find a significant longer-term reduction in serious violent crimes. Although it is unclear whether these reductions are attributed to a long-term effect of the programme itself, or due to specific factors relating to the COVID-19 pandemic, our findings highlight significant potential of a combined public health and criminal justice approach at the municipal-level. Continued monitoring of the effects of such interventions is critical for understanding their role in reducing violence in cities around the world.

## Contributors

MDE and JM conceptualised and designed the study. MDE wrote the study protocol, and CC, EVS and JM contributed to the protocol. MDE and EVS collected the quantitative data for analysis, and CC, EVS, DB, and ER collected the qualitative data via stakeholder interviews. MDE did the data analysis, with support from AGS and IC. MDE drafted the manuscript, and all authors participated in data interpretation and critical review of the manuscript. JM supervised the project throughout. All authors read and approved the final manuscript. All authors were able to gain full access to the data, and MDE and JM were responsible for the decision to submit the manuscript. All authors had the final responsibility for the decision to submit for publication.

## Data sharing statement

The data in this manuscript represent publicly accessible routinely collected data, including criminal and mortality statistics (see methods and references for information and corresponding links). We have further made the data used in the analyses openly and easily available at the Open Science Framework (https://osf.io/3xaew/).

## Declaration of interests

JM collaborated with the Municipal Government of Pelotas on one project included in the Pelotas Peace Pact. That was a randomised trial of two of the early childhood parenting interventions, as documented in Murray et al. (2019),^43^ and the municipal government contributed funds to conduct the trial. All other authors declare no competing interests.

## Appendix A Supplementary data

Webappendix
