# Supplementary material for: Effects of the Pelotas (Brazil) Peace Pact on violence and crime: a synthetic control analysis
Source: Lancet Reg Health Am. 2023 Feb 21;19:100447. doi: 10.1016/j.lana.2023.100447 (PMC9982028; doi:10.1016/j.lana.2023.100447)
Supplement: Webappendix [file mmc1.docx]

**Effects of the Pelotas (Brazil) Peace Pact on violence and crime: a synthetic control analysis –** Degli Esposti *et al*

**Web-appendix**

| **Contents** | | **Page** |
| --- | --- | --- |
| **Web-text** | | |
|  | Web-methods: Stakeholder interviews | 3 |
|  | Web-methods: Advantages and challenges of synthetic control methodology | 6 |
|  | Web-methods & results: Technical summary of synthetic control methodology | 6 |
| **Web-tables** | |  |
|  | Web-table 1: Summary of five main axes of the Pelotas Pact for Peace (“Pacto”) | 7 |
|  | Web-table 2: Methodological details on the interviews with executive secretaries of the Pelotas Pacto for Peace and selected programme coordinators in Social Prevention Axis | 8 |
|  | Web-table 3: Summary of Social Prevention Axis projects of the Pelotas Pact for Peace | 9 |
|  | Web-table 4: Methodological details on the interviews with representatives of institutions in Policing and Justice Axis | 12 |
|  | Web-table 5: Standardised criminal definitions | 13 |
|  | Web-table 6: Summary of outcome variables | 14 |
|  | Web-table 7: Summary of control variables (hypothesised confounders) | 15 |
|  | Web-table 8: Summary of separate synthetic control models for evaluating the effects of Pelotas Pact for Peace, before and during the COVID-19 pandemic | 16 |
|  | Web-table 9: Counts before and after the Pelotas Pact for Peace and Focussed Deterrence in Pelotas and 22 control municipalities in Rio Grande do Sul | 17 |
|  | Web-table 10: Average rates before and after the Pelotas Pact for Peace and Focussed Deterrence in Pelotas and 22 control municipalities in Rio Grande do Sul | 18 |
|  | Web-table 11: Weights for synthetic controls for evaluating the effects of Pelotas Pact for Peace | 19 |
|  | Web-table 12: Average covariate balance before Pelotas Pact for Peace was introduced for Pelotas and the synthetic controls, police- and health-recorded homicide | 20 |
|  | Web-table 13: Average covariate balance before Pelotas Pact for Peace was introduced for Pelotas and the synthetic controls, property crime | 21 |
|  | Web-table 14: Effects of Pelotas Peace Pact on health-recorded homicide before and during the COVID-19 pandemic, compared with synthetic controls | 22 |
|  | Web-table 15: Placebo tests and robust t-tests estimating the significance of the effects of Pelotas Peace Pact, before and during the COVID-19 pandemic | 23 |
|  | Web-table 16: Average covariate balance before Pelotas Pact for Peace was introduced for Pelotas and the synthetic controls, yearly outcomes | 24 |
|  | Web-table 17: Sensitivity analysis for the effects of Pelotas Peace Pact on monthly violence and crime, controlling for monthly COVID-19 measures (COVID-19 related deaths and google mobility) | 25 |
|  | Web-table 18: Sensitivity analysis for the effects of Pelotas Peace Pact on monthly violence and crime, pre-filtering the monthly outcome series | 26 |
|  | Web-table 19: Sensitivity analysis for the effects of Pelotas Peace Pact on crime, violence, and school dropout, excluding Porto Alegre from the donor pool | 27 |
| **Web-figures** | |  |
|  | Web-figure 1: A schematic logic model of the effects of the Pelotas Peace Pact violence and crime | 28 |
|  | Web-figure 2: A schematic diagram of the proposed strands and targeted interventions delivered under the Social Prevention Axis | 29 |
|  | Web-figure 3: A schematic illustration of the proposed strands and targeted interventions delivered under the Policing and Justice Axis | 30 |
|  | Web-figure 4: A schematic illustration of the proposed strands and targeted interventions delivered under Administration, Urbanism, and Technology Axes | 31 |
|  | Web-figure 5: A map of municipalities in Rio Grande do Sul, highlighting Pelotas (treated unit) and a donor pool of 22 potential control units (with population sizes ≥80,000 and without similar city-wide interventions [Canoas, Lajeado]) | 32 |
|  | Web-figure 6: Size of the estimated (placebo) effects of the Pelotas Pact for Peace on police-recorded homicide standardized by pre-intervention fit in all municipalities (ordered from largest to smallest), for the full post-intervention period | 33 |
|  | Web-figure 7: Size of the estimated (placebo) effects of the Pelotas Pact for Peace on police-recorded homicide standardized by pre-intervention fit in all municipalities (ordered from largest to smallest), before and during the COVID-19 pandemic | 34 |
|  | Web-figure 8: Size of the estimated (placebo) effects of the Focussed Deterrence Strategy on police-recorded homicide standardized by pre-intervention fit in all municipalities (ordered from largest to smallest), for the full post-intervention period | 35 |
|  | Web-figure 9: Size of the estimated (placebo) effects of the Focussed Deterrence Strategy on police-recorded homicide standardized by pre-intervention fit in all municipalities (ordered from largest to smallest), before and during the COVID-19 pandemic | 36 |
|  | Web-figure 10: Size of the estimated (placebo) effects of the Pelotas Pact for Peace on robbery standardized by pre-intervention fit in all municipalities (ordered from largest to smallest), for the full post-intervention period | 37 |
|  | Web-figure 11: Size of the estimated (placebo) effects of the Pelotas Pact for Peace on robbery standardized by pre-intervention fit in all municipalities (ordered from largest to smallest), before and during the COVID-19 pandemic | 38 |
|  | Web-figure 12: Size of the estimated (placebo) effects of the Pelotas Pact for Peace on theft standardized by pre-intervention fit in all municipalities (ordered from largest to smallest), for the full post-intervention period | 39 |
|  | Web-figure 13: Size of the estimated (placebo) effects of the Pelotas Pact for Peace on theft standardized by pre-intervention fit in all municipalities (ordered from largest to smallest), before and during the COVID-19 pandemic | 40 |
|  | Web-figure 14: Size of the estimated (placebo) effects of the Pelotas Pact for Peace on vehicle theft and robbery standardized by pre-intervention fit in all municipalities (ordered from largest to smallest), for the full post-intervention period | 41 |
|  | Web-figure 15: Size of the estimated (placebo) effects of the Pelotas Pact for Peace on vehicle theft and robbery standardized by pre-intervention fit in all municipalities (ordered from largest to smallest), before and during the COVID-19 pandemic | 42 |
|  | Web-figure 16: Controlled interrupted time series estimates for the effect of the Pelotas Peace Pact and Focussed Deterrence on police-recorded homicide, compared with synthetic controls, before and during the COVID-19 pandemic | 43 |
|  | Web-figure 17: Controlled interrupted time series estimates for the effect of the Pelotas Peace Pact on property crime, compared with synthetic controls, before and during the COVID-19 pandemic | 44 |
| **Web-references** | | 45 |

***Web-methods & results*: Stakeholder interviews**

All interviews were previously approved by the Research Ethics Committee of the Federal University of Pelotas School of Medicine (CAAE registration number: 53190721.0.0000.5317).

**Social Prevention Axis**

On the 24^th^ of March 2022, we conducted an interview with executive secretariat of the Pelotas Pact for Peace (“Pacto”) which included the participation of the former and the new secretaries (from March 2020) of the Pacto. The main aim of this interview was to gather more detailed information about the scope of all projects that are part of the Social Prevention Axis of the Pacto, and update on any changes that were caused by the COVID-19 pandemic. The methodology of the interviews are described in web-table 2. We collated, confirmed, and summarised the information gathered about the Social Prevention Axis projects in web-table 3.

In addition to this information, we asked them to name the projects most strongly implemented according to their perception. The following question was used “Within the social prevention axis, for each level (primary, secondary, and tertiary prevention), which programs do you consider having been implemented most strongly to date?”. Consensus responses were obtained, and these projects are listed below.

- Primary prevention:
  - Raising Safe Kids (ACT) had a very involved coordination that embraced the program with great affection and energy. This is fundamental for the development and multiplication of initiatives. Today, all kindergarten schools and some elementary schools have facilitators.
- Secondary prevention:
  - Every Youth Counts, Opportunities Map and Start were complementary effective strategies working to reduce school-drop out – although the coverage of schools still limited.
- Tertiary prevention:
  - The Association for the Protection and Assistance to the Convicted (APAC) is the most significant project to re-integrate prisoners.

Afterwards, project coordinators from the above programmes were invited and interviewed with the purpose of proving more details about their implementation and changes during the COVID-19 pandemic. These interviews were semi-structured and guided by topic questions. Most interviews were done through video conference and length from 45 to 60 minutes, see web-table 2.

The qualitative work highlighted the following overall aspects of the Social Prevention Axis programmes’ implementation:

- The Pacto covers a wide range of Social Prevention programmes, which were mainly implemented and delivered across the education, health, and social assistance sectors. Their aims, target population and coverage were vast scope and cut across primary, secondary, and tertiary prevention. It should be noted, however, that a handful of the programmes that are currently part of Pacto were implemented before its beginning (August 2017) but were strengthened (e.g., Better Early Childhood and Happy Child) as part of the Pacto’s implementation.
- Coordinators of each programme engaged in parallel monthly meetings with the executive secretariat of Pacto as part of the activities of the “Integrated Prevention Committee”. These meetings happened from the beginning of Pacto until March 2020, when the sectors of education, health and social assistance switched their focuses to give full support to the public health actions to deter the impacts of the COVID-19 pandemic.
- The “Integrated Prevention Committee” is seen as a key component of the Pacto. For example, the programme “Each Youth Count” targets secondary prevention, identifying and referring adolescents at risk of school dropout to other programmes such as “Opportunities Map”, “START”, and “Sports for Peace”. An integration of processes and a shared agenda between the programmes is therefore needed to allow for such cross-programme communication.
- The lack of pre-specified financial resources for each programme actions and the fact that activities generated an extra workload for each department, suggested that the programmes relied on their coordinators' personal motivation and ability to efficiently lead action. One example of lead action was shared by the former coordinator of “Opportunities Map”. The program referred young people for the job vacancies, but the programme leads also identified a need to train young people to give them the strongest footing for retaining these job opportunities. As a result, the “START” programme was created with the aim to first train young people, and then refer them to the job vacancies. For this reason, a 2-month course of preparation for the job market was created.
- A limitation reported by interviewees was the lack of data and valid indicators to allow monitoring of the progress and impact of most programmes. The coordinators of “Each Youth Count” programme, for example, indicated improvements in school attendance in the pre-pandemic period as a positive result but highlighted that this was only based on anecdotal evidence – the perceptions of education coordinators. It was only recently (at the beginning of 2022) that the programme developed a new information system to longitudinally follow adolescents in the programme. In this new system, case management and referrals to health, sports activities, and opportunities in the job market are carried out, as well as the generation of panels with school attendance and automated reports.
- According to the programme coordinators, some changes and refinements to the target population occurred over time. For example, initially school coordinators were responsible for participant selection into the “ACT” programme. As of 2022, the program began to serve the entire school network in the municipality and prioritize cases identified based on the criteria of low attendance and school dropout. Priority is also given to the children of prisoners, students with greater vulnerability, those with poor school performance, and students who did not return after the pandemic. For the “Opportunities Map”, the focus was originally exclusively on vulnerable young people. However, because programme adherence was low in 2018, the coordinator adapted the classrooms dynamics, which in turn diversified the target population, mixing more and less vulnerable young people.
- The COVID-19 pandemic stopped almost all the programme activities in the first half of 2020. With the interruption of in-person classes at schools, most social prevention programmes stopped their activities. While there were some attempts to implement virtual activities, adherence to these virtual activities was very low. During 2022 there was a gradual recovery of the activities, but some programmes have still not completely resumed.
- According to the interviewees, the strong multi-sectoral work among institutions was a positive and innovative component of Pacto, which is viewed as a legacy of the intervention.

**Police and Justice Axis**

Additional interviews were conducted with key actors from the police, judiciary, and prison systems to update on activities part of the Police and Justice Axes. The methodological detail of the interviews with these different actors is outlined in web-table 4.

The Pacto integrates various institutional actors of the Criminal Justice System to enact repressive and preventive strategies to reduce crime. The Pacto's Policing and Justice axis is led by the Mayor through the Municipal Integrated Management Office, which has active participation of the Civil Police, the Military Police, the Federal Police, the Public Ministry, the Judiciary, the Fire Department, the Municipal Guard, and the Penitentiary System. Every month, representatives of these institutions keep meetings to define priorities and actions oriented to improve public security in Pelotas. Specifically, the Municipal Public Security Observatory has a central role in this joint working process, because it prepares technical reports for monitoring and developing evidence-based initiatives. Focused Deterrence and hotspots policing were two notable interventions, but coordination also improved the work of the institutions more broadly.

The qualitative work carried out highlighted the following:

- The action and coordination between criminal justice institutions represents an improvement in strategic planning. According to most interviewees, the integration between different institutions of the criminal justice system was primarily responsible for the increase of public security and allowed Pelotas to better face the unexpected health crisis derived from COVID-19. However, the interviewees also reported that there is still a lack of integration between the Policing and Justice and Social Prevention Axes.
- According to the interviewees, the Focused Deterrence strategy was the primary reason for the observed reduction in homicide, although it ended up being discontinued in March 2020. Phasing out of the strategy started because of the initiative's success and because the local reality changed: violent crime had dropped and taken on different characteristics after the disintegration of some criminal gangs and the end of a factional war that took place until 2019. In fact, since 2020 there is a monopoly of a single gang for which street violence is not desirable because it draws the attention of the authorities and hinders illegal business. With relatively low levels of homicide and no conflict between factions, the Focused Deterrence strategy was no longer considered necessary by authorities.
- The government of the state of Rio Grande do Sul created “RS Seguro” in February 2019 (Decree 54,516), a programme that aims to integrate public security, education, health, sport, leisure, work, social assistance, culture, justice, and social development. The initiative has four axes: i) combating crime; ii) social, preventive and transversal policies; iii) qualification of service to citizens; and iv) prison system. The programme has a territorial focus and was applied in the 23 municipalities in the state with the highest incidence of violent crimes: Alvorada, Bento Gonçalves, Cachoeirinha, Canoas, Capão da Canoa, Caxias do Sul, Cruz Alta, Esteio, Farroupilha. Gravataí, Guaíba, Ijuí, Lajeado, Novo Hamburgo, Passo Fundo, Pelotas, Porto Alegre, Rio Grande, Santa Maria, São Leopoldo, Sapucaia do Sul, Tramandaí, Viamão. The Pact and RS Seguro are separate programmes, but with similar objectives.

On the effects of COVID-19, respondents reported that the pandemic reduced crime, especially crimes against property due to less urban mobility. Additionally, the health emergency affected the performance of the institutions. In the period from 2020 to mid-2021, there was an intensification of integrated operations, that began to monitor compliance with measures to combat the pandemic, defined in municipal law 6,261 published in April 2020. The municipality was able to take advantage of pre-existing institutional network for health inspections and indirectly strengthen public security. This is because, while mobility was reduced, there were also more police on the streets. On the other hand, the pandemic also affected serving sentences: the population with health risks and all people imprisoned in a semi-open regime were placed under house arrest, with electronic monitoring, to prevent the spread of the virus.

**Administration, Urbanism & Technology Axes**

We were informed from the main interview with the secretariat that projects from the Administration, Urbanism and Technology Axes were under development. As such, these projects were not implemented by the end of 2021. For this reason, no interviews were conducted for the Administration, Urbanism and Technology Axes.

***Web-methods*: Advantages and challenges of synthetic control methodology**

SCM has several advantages over more conventional evaluation methods for situations where randomised controlled trials are difficult to realise (such as city-level interventions), including difference-in-differences (DiD) and interrupted time series analyses (ITS). First, it reduces researcher bias by using a data-driven optimisation approach to estimate the counterfactual in a transparent way. Second, it simultaneously accounts for both differences in time-invariant characteristics between treated and control units and from confounding factors that vary over time. Third, SCM offers a flexible approach for evaluating the shape and magnitude of the intervention effect over time since the method relies on weaker identification assumptions.

However, a challenge of SCM is obtaining valid inference statistics (p-values and confidence intervals) due to serial correlation, regularisation bias from non-normal sampling distributions of the effect estimates, and only having a single treated unit.^1^ To facilitate statistical inference, we performed three extensions: tests of difference-in-differences, placebo tests, and robust t-tests. The former inference method was prioritised due to its ability to test and account for imperfect synthetic control fit and is described in the manuscript. Here we described the second two approaches we used to help assess the significance of the effects of the Pacto.

Our first supplementary approach used placebo tests to compute quasi *p* values.^2^ These are calculated via artificially assigning hypothetical interventions that “occur” at the same time as the Pacto in each municipality in the donor pool and estimated effects using SCM. These so-called “placebo effects” are then compared to the estimated effect of the Pacto. If the intervention effect identified in Pelotas (n=1) is larger than all placebo effects (n=22) with comparable pre-intervention fit (e.g., quasi *p* value: 1/23= 0.043), then this indicates statistical significance; on the other hand, if the effect in Pelotas is smaller than comparable placebo-effects (e.g., quasi *p* value: 10/23= 0.435), then this indicates the estimated effect of the Pacto is likely due to chance. We plotted ordered bar charts of the intervention effect in Pelotas and all placebo effects, while standardising by synthetic control fit, to visualise the likelihood that the effect of Pacto was identified by chance.

Our second supplementary approach used robust t-tests to derive bias-corrected effect estimates and corresponding 95% confidence intervals (CI).^3^ This method draws from literature on statistical inference for machine learning estimators to adjust for regularisation bias, recovering a normal sampling distribution. The method creates synthetic controls while holding out different portions of the pre-intervention data before the intervention for cross-validation and estimating the regularisation bias. Specifically, the mean difference between the outcomes in Pelotas and the synthetic controls in the holdout periods estimates the bias. This bias is then subtracted from the effect estimates and confidence intervals are constructed using a t-distribution.

***Web-methods*: Technical summary of synthetic control construction**

SCM uses pre-intervention outcomes and covariates as input for the balancing procedure (i.e., matching process). A nested optimisation algorithm is then applied to identify the optimal set of (time-invariant) unit weights, see elsewhere for mathematical equations.^1,2^ The algorithm takes the vector of auxiliary pre-intervention covariates and some combination of pre-intervention outcomes to determine the weights. The optimal unit weights are found by minimizing the (variable-importance weighted) root mean squared error between the observed data in the treated unit and the synthetic control. In other words, the weights used to derive the synthetic control depend on the identified variable importance weights – in our analyses this includes all listed confounders and the outcome under evaluation (web-tables 6 & 7).

***Web-methods*: Health-recorded homicide**

To check the robustness of our findings across data sources, we analysed monthly rates of health-recorded homicide (i.e., mortality by relevant cause of death) by municipality from January 2012 to December 2020 (latest available data at time of analysis). This was conceptualised as a sensitivity analysis due to a lack of data availability for 2021 at the time of analysis. We extracted mortality data by cause of death from the Brazilian Ministry of Health's Mortality Information System (SIM).^4^ To match the criminal definition of homicide, health-recorded homicides were defined as external causes of death by another person with an intent to injure or kill according to the International Classification of Diseases (10th revision) (ICD-10) codes: X85-Y09 for assault.

***Web-table 1*: Summary of five main axes of the Pelotas Pact for Peace (“Pacto”)**

| **Axis 1: Social Prevention** | |
| --- | --- |
|  | Primary and secondary programmes focusing on promoting children and young people and tertiary programmes for incarcerated youth |
| **Axis 2: Policing and Justice** | |
|  | Attempts to introduce proactive and integrated evidence-based strategies to improve security and justice |
| **Axis 3: Administration** | |
|  | Consists of a set of inspections conducted by the municipality to avoid disturbing peace and small crimes |
| **Axis 4: Urbanism** | |
|  | Aims to implement urban initiatives and regulations to promote a sense of security and reduce violence in the city |
| **Axis 5: Technology** | |
|  | Aims to support and facilitate the introduction of new technology to measure and monitor violence and crime in the city |

***Web-table 2:* Methodological details on the interviews with executive secretaries of the Pelotas Pacto for Peace and selected programme coordinators in Social Prevention Axis**

| **Pelotas Pact for Peace** | **Date** | **Duration** | **Interviewers** | **Respondent** | **Medium** |
| --- | --- | --- | --- | --- | --- |
| Executive secretaries | Mar 24, 2022 | 120 min | EVS, CC, JM, MDE, DB and ER | Current and former executive secretaries | Hybrid (most people in person) |
| **Programme (English / Portuguese name)** |  |  |  |  |  |
| Opportunities Map / Mapa de Oportunidades | May 17, 2022 | 45 min | EVS and CC | Former programme coordinator | Video conference |
|  | May 26, 2022 | 60 min | EVS | Current programme coordinator | In person |
| Every Youth Counts / Cada Jovem Conta | May 25, 2022 | 60 min | EVS and CC | Current and former programme coordinator | Video conference |
| START / START | May 17, 2022 | 45 min | EVS and CC | Current programme coordinator | Video conference |
| Raising Safe Kids (ACT) / Educar Crianças em Ambientes Seguros | May 30, 2022 | 50 min | EVS | Current programme coordinator | Video conference |
| **Questions used to introduce topics in semi-structured interviews with programme coordinators** | | | | | |
| 1. When did the programme start? | | | | | |
| 2. What are the current objectives of the programme? Have they changed over time? | | | | | |
| 3. Which institutions participate and how are they articulated? | | | | | |
| 4. What human and financial resources are available for this project? | | | | | |
| 5. What is the curriculum of the activities and where are they developed? | | | | | |
| 6. Has this curriculum changed over time? | | | | | |
| 7. How can these activities help to prevent violence? | | | | | |
| 8. How many people have participated in the programme? What is the profile of the beneficiaries? | | | | | |
| 9. How has the COVID-19 pandemic affected the development of programme activities? | | | | | |
| 10. How are the results monitored? What are the results obtained so far? | | | | | |
| 11. What are the main limitation and the main strength to implement the programme? | | | | | |

***Web-table 3:* Summary of Social Prevention Axis projects of the Pelotas Pact for Peace**

| **Strategy name** | | **Projects by prevention level (English / Portuguese names)** | **Aim** | **Start date** | **Target population** | **Number of beneficiaries** | **Status during the COVID-19 pandemic** | |
| --- | --- | --- | --- | --- | --- | --- | --- | --- |
|  |  |  |  |  |  |  | **Interrupted in March 2020** | **Date resumed** |
| **Primary Prevention:** To prevent violence in the family and school environment | | | | | | | | |
|  | School of Peace | School of Peace / Escola da Paz | Build a culture of peace by monitoring school violence. Create Internal Committees for the Prevention of School Violence | Mar 2018 | Students attending public schools | 22791 | Yes | Jul 2021 |
|  |  | Socioemotional Education / Educação Socioemocional | Promote Socio-Emotional Education, including self-awareness of different emotions and offers a better approach to addressing conflicts in the school environment | Feb 2020 | Public schools | 30 teachers and 1354 participants in 5 schools | Yes |  |
|  |  | Building Knowledge / Construindo Saberes | Provide conditions for underachieving students to overcome difficulties related to the learning process and prevent school drop-out | Aug 2016* | Students with a school delay of at least two years | 75 students and 3 teachers | Yes |  |
|  | Sports for Peace | Gymnastics / Ginástica Artística | Offer training and practices for gymnastics and promote body and mental health | Apr 2017 | Students attending public schools | ~200 children (5 to 15 years) | Yes | Not resumed |
|  |  | Parasports / Paradesporto | Encourage parasports and practice adapted sports among people with physical, visual, and intellectual disabilities | Dec 2019 | People with physical, visual and intellectual disabilities | 65 | Yes | Mar 2022 |
|  |  | Athletics / Atlestismo | Offer the practice of Athletics in the public education network, through partnerships with schools and neighbourhood associations | Apr 2014* | Students attending public schools | 145 (11 to16 years) | Yes | Mar 2022 |
|  |  | Martial Arts / Quem Luta Não Briga | Offer the practice of Taekwondo, Olympic Combat, from sports initiation to high performance | Mar 2011* | Students attending public schools | 520 | Yes | Mar 2022 |
|  |  | Volleyball / Voleibol | Provide opportunities for learning volleyball, aiming to foster social inclusion through sports practice | Sept 2014* | Children and youth (10 to 17 years) | 2000 | Yes | Mar 2022 |
|  |  | Active Life / Vida ativa | Encourage leisure, sport, and physical activity for all ages | Sept 2013* | General population | 3000 | .. | .. |
|  | Restorative Justice | Good Neighbours / Bons Vizinhos | Conflict prevention and resolution to improve co-living in Popular Residential Condominiums | Dec 2017 | Residents of popular housing condominiums | No information | Yes | Not resumed |
|  |  | Peacebuilding Circles / Círculos de Construção da Paz | Prevent violence in schools and public services by training facilitators that develop peacebuilding circles | Aug 2017 | Public schools and services at the municipal level | Circles: 952 Participants: 14831 Schools: 58 Facilitators: 175 | No | .. |
|  | Protected Childhood | Strong Families / Famílias Fortes | Strengthen family ties to prevent risky adolescent behaviours (e.g., drug use) | Dec 2021 | Youth (10 to 14 years) | 15 facilitators | .. | .. |
|  |  | Count on Me (Dialogic Book Sharing) / Conte Comigo | Ensure the socio-cognitive and emotional development of children through book sharing tasks between parents/children | Jun 2018 | Children (3 to 6 years) | 200 families and 100 children | Yes | Mar 2021 |
|  |  | Raising Safe Kids (ACT) / Educar Crianças em Ambientes Seguros | Train and educate parents and other caregivers away from any violence and encourage good parenting practices, such as strengthening family bonds | Jul 2018 | Children (0 to 6 years) | 500 | Yes | Mar 2021 |
|  |  | Better Early Childhood (PIM) and Happy Child / Primeira Infância Melhor e Criança Feliz | Support families based on their culture and experiences and promote the development of children | Mar 2003* | Pregnant women and children (0 to 6 years) | 1173 | No | .. |
|  |  | Early Pregnancy Monitoring / Programa de Prevenção à Gravidez Precoce | Carry out preventive actions against early pregnancy, including data gathering and monitoring early pregnancy and ensuring access to prenatal care and school attendance | Dec 2017 | Youth (10 to19 years) | No information | Yes | Mar 2022 |
|  |  | Eradication of Sub-Civil Birth Registration / Erradicação do Sub-registro Civil de Nascimentos | Interconnect the municipal maternity hospitals with the civil registry offices | Dec 2017 | New-borns | No information | Yes | Not resumed |
|  |  | Reduction of Early Victimization / Redução da Vitimização Precoce | Actions to strengthen the protection network for children and adolescents (e.g., qualification of services flows and protocols for victims of violence). | Dec 2017 | Directors of services and institutions involved in the protection network | ~ 5 meetings involving employees capacitation | No | .. |
|  |  | Start Well / Começar Bem | Strengthen the entrepreneurial spirit through workshops, courses, and lectures | Jun 2019 | Youth and adults attending public schools | 60 | Yes | Not resumed |
|  |  | Young Entrepreneurs First Steps / Jovens Empreendedores Primeiros Passos | Enable learning and development of entrepreneurial skills and behaviours | Feb 2017 | Students attending public schools | 4395 students and 82 teachers | Yes | Not resumed |
|  | School of Peace | Active School Search / Busca Ativa Escolar | Identifies children and adolescents out-of-school and seeks to guarantee their return, with monitoring of the network to maintain attendance | Mar 2021 | Out-of-school youth | 137 | .. | .. |
| **Secondary Prevention:** To reduce at-risk youths’ involvement in violence and crime | | | | | | | | |
|  | Every Youth Counts | Every Youth Counts / Cada Jovem Conta | Reduce risk factors for violence against children and adolescents in Elementary School, especially school dropout. | Sept 2017 | At risk youth from the most violent areas | 340 | Yes | Aug 2021 |
|  |  | START/START | Train, qualify, and create spaces for vulnerable youth to access work, by encouraging protagonism, leadership, autonomy, and entrepreneurship | Jun 2018 | Youth (14 to 22 years) | 600 | Yes | Jun 2021 |
|  |  | Opportunities Map / Mapa de Oportunidades | Seek job opportunities, income generation and training (captures, organizes and map job vacancies obtained through partnerships between public, private, civil society institutions) | Oct 2017 | Youth, Women in Vulnerable Situations, Migrants, Monitored and Egressed from the Prison System | 1000 | Yes | Jan 2022 |
|  |  |  |  |  |  |  |  |  |
|  |  | Decent Housing / Moradia Digna | Carries out housing improvements and offers social support for vulnerable families participating in “Every Youth Accounts” programme to facilitate school attendance | Oct 2021 | Youth who have risk factors for violence and are participating in Every Youth Counts | 1 house (11 more to come) | No | .. |
|  |  | Youth Space / Espaço da Juventude | Promote the integration of young people into the community, developing their autonomy and leadership to overcome their disadvantage | Jul 2019 | Youth from vulnerable communities | No information | Yes | Not resumed |
| **Tertiary Prevention:** To reduce criminal recidivism among youth and adults | | | | | | | | |
|  | Second Chance | Cultural Workshops for Socio-Education / Oficinas Culturais na Socioeducação | To promote cultural workshops for adolescents to help them and carve out a meaningful future life plan | Aug 2017 | Juvenile offenders | No information | Yes | Mar 2022 |
|  |  | Association for the Protection and Assistance to the Convicted / Associação de Proteção ao Condenado (APAC) | Offers opportunities to help reintegrate offenders during their sentence by emphasising and developing values and skills related to education, work, and spirituality | May 2020 | Adult offenders in jail (closed regime) | 20 | No | .. |
|  | Prison Labour | Public Health System / Sistema Único de Saúde (SUS) | Reintegration through work: Renovating buildings of the Public Health System | Sep 2015* | Adult offenders released from jail (electronic monitoring) | 16 | No | .. |
|  |  | Urban Services / Serviços Urbanos | Reintegration through work: General urban cleaning services, drainage, and qualification of unpaved roads | Aug 2016* | Adult offenders released from jail (electronic monitoring) | 31 | No | .. |
|  |  | Construction and Paving / Obras e Pavimentação | Reintegration through work: Laying and repair of paving, curb, asphalt on paved public roads | Feb 2021 | Adult offenders released from jail (electronic monitoring) | 32 | No | .. |
|  |  | Social Assistance / Assistência Social | Reintegration through work: Providing support in general services | Aug 2018 | Adult offenders released from jail (electronic monitoring) | 6 | No | .. |
|  |  | ArtconP / ArtconP | Reintegration through work: Manufacturing of concrete products inside the facilities of the regional prison of Pelotas | Jul 2019 | Adult offenders in jail (closed regime) | 10 | No | .. |

The table summarises all Social Axis Prevention Projects that were implemented until November 2021 (see web-figure 1 for the corresponding logic model.

*Project implemented before Pacto but the Pacto developed and expanded these programmes further.

***Web-table 4:* Methodological details on the interviews with representatives of institutions in Policing and Justice Axis**

| **Institution** | **Date** | **Duration** | **Interviewers** | **Respondent** | **Medium** |
| --- | --- | --- | --- | --- | --- |
| Municipal Security Observatory | April 22, 2022 | 40 min | DB | Coordinator | Video conference |
| Court of Criminal Executions | April 19, 2022 | 50 min | ER | Judge | Video conference |
| Penitentiary system | April 20, 2022 | 45 min | ER | Delegate | Video conference |
| APAC (a rehabilitation initiative) | April 20, 2022 | 45 min | ER | President | Video conference |
| Military Police | April 22, 2022 | 55 min | DB | Former Battalion Commander in Pelotas | Video conference |
| Civil Police | April 25, 2022 | 50 min | DB | Civil Police Delegate | Video conference |
| Court of Criminal Executions | May 02, 2022 | 45 min | ER | Former Judge | Video conference |
| Municipal Security Department | May 02, 2022 | 40 min | DB | Secretary | Video conference |
| Military Police | May 02, 2022 | 45 min | DB | Battalion Commander in Pelotas | Video conference |
| **Questions used to introduce topics in-semi structured interviews** | | | | | |
| 1. What are the current goals of the Pacto’s Policing and Justice axis? Have these goals changed since 2019? | | | | | |
| 2. Which institutions currently participate in the Policing and Justice axis? How do they link up with each other? | | | | | |
| 3. What are the activities developed within the Policing and Justice axis of the Pacto? Have these activities changed since 2019? | | | | | |
| 4. What is the participation of your institution in the Pacto? / Has this share changed since 2019? | | | | | |
| 5. Had any change in the ostensible patrolling of the Military Police since 2019? | | | | | |
| 6. Had any change in the Civil Police investigation of homicides since 2019? | | | | | |
| 7. Were there any transfers of prisoners or threats of transfers of prisoners due to street homicides in 2020 and 2021? Have there been changes to this policy since 2019? | | | | | |
| 8. What have been the results obtained so far by the Pacto? Have the results changed since 2019? | | | | | |
| 9. Did the COVID-19 pandemic directly affect the dynamics of violence and crime in Pelotas? | | | | | |
| 10. Did the COVID-19 pandemic affect the development of activities in the Policing and Justice axis of the Pacto? | | | | | |

***Web-table 5*: Standardised criminal definitions**

| **Homicide (Intentional Lethal Violent Crimes)** | |
| --- | --- |
|  | Number of homicide victims; homicide will be defined as all wrongful occurrences of deaths from intentional lethal violent crime (CVLIs). As per norms in Brazil, these include homicide (including in motor vehicle management), assault (bodily injury) and robbery resulting in death, abortion, infanticide, feminicide, assisted suicide, torture resulting in death, rape resulting in death, and extortion through kidnapping resulting in death.^5^ |
| **Crimes Against Property** | |
|  | Number of occurrences of all robberies and thefts, as well as vehicle robbery and theft:   - ***Robbery (all)*** the stealing of someone else's movable property, for oneself or for others, through serious threat or violence to the person, or after having it, by any means, keeping possession through the use of force. - ***Theft (all)*** stealing for yourself, or for others, something that is mobile not involving the use of force. - ***Vehicle robbery & theft*** stealing using threat or violence (robbery), or otherwise (theft), a motor vehicle. |
| **Violence Against Women** | |
|  | Number of female victims of assault; defined as an offence on a woman’s bodily integrity (including mild bodily injury) and/or her health. |

For homicide the unit of analysis is the number of victims and for crimes against property and women the unit of analysis is the number of occurrences of the crime, given this is how they are recorded by state officials.

***Web-table 6*: Summary of outcome variables**

| **Category** | | **Outcome** | **Unit of analysis** | **Time period (intervals)** | **Conceptualisation of outcome under evaluation** | **Notes** |
| --- | --- | --- | --- | --- | --- | --- |
| **Police-recorded crime, Secretary for Public Security**^6^ | | | | | | |
|  | Violence against person | Homicide (Intentional Lethal Violent Crimes, CVLI) | Person | Jan 2012 – Dec 2021 (monthly) | Primary outcome in the evaluation of: i) Pelotas Pact for Peace, Aug 2017; and ii) Focussed Deterrence, May 2018 | Feminicide was included in this criminal category up until 2018, after which it was recorded in the separate criminal category of feminicide. Consultations with the Secretariat confirmed that these numbers are very small are unlikely to significantly affect the consistency of the data over the study period. |
|  | Crimes against property | Robbery (all) | Occurrence | Jan 2012 – Dec 2021 (monthly) | Secondary outcome in the evaluation of Pelotas Pact for Peace, Aug 2017 | There was a temporal delay in the full reporting of property crime. This was particularly true for theft where figures were often updated over 6 months after the fact. |
|  |  | Theft (all) | Occurrence | Jan 2012 – Dec 2021 (monthly) | Secondary outcome in the evaluation of Pelotas Pact for Peace, Aug 2017 | There was a temporal delay in the full reporting of property crime. This was particularly true for theft where figures were often updated over 6 months after the fact. |
|  |  | Vehicle robbery and theft (combined) | Occurrence | Jan 2012 – Dec 2021 (monthly) | Secondary outcome in the evaluation of Pelotas Pact for Peace, Aug 2017 | There was a temporal delay in the full reporting of property crime. This was particularly true for theft where figures were often updated over 6 months after the fact. |
|  | Violence against women | Assault (including mild) | Occurrence | 2012 – 2021 (yearly) | Tertiary outcome in the evaluation of: i) Pelotas Pact for Peace, Aug 2017; and ii) Focussed Deterrence, May 2018 | Data are only available yearly and by occurrence of crime during the study period. However, consultations with the Secretariat revealed that over 90% of all occurrences only included one victim. |
| **Health data, Brazilian Ministry of Health**^7,8^ | | | | | | |
|  | Mortality due to external causes by another person | Mortality due to assault (ICD-10: X85-Y09) | Person | Jan 2012 – Dec 2020 (monthly) | Robustness check of police-recorded CVLI (the primary outcome in the main evaluation of Pelotas Pact for Peace and Focussed Deterrence) | .. |
| **School Data, Secretary for Education**^9^ | | | | | |  |
|  | School dropout | Student dropout from elementary (Ensino Fundamental) and middle school (Ensino Medio) | Student | 2014 – 2021 (yearly) | Tertiary outcome in the evaluation of the Pelotas Pact for Peace, Aug 2017, that is hypothesised to be impacted by interventions delivered under the Social Prevention Axis. | .. |

While the study period spanned from Jan 2012 until July 2021, not all data were available for the full time period. For example, consistently collected health data were only available from Jan 2014.

***Web-table 7*: Summary of control variables (hypothesised confounders)**

| **Confounder** | **Measure** | **Region** | **Time period** | **Source** |
| --- | --- | --- | --- | --- |
| Population sociodemographic | Municipal population age sex-race distributions | Municipality | 2010 | Census, The Brazilian Institute of Geography and Statistics (Instituto Brasileiro de Geografia e Estatística, IBGE) |
| Economic | % of population living in poverty | Municipality | 2010 | Census, The Brazilian Institute of Geography and Statistics (Instituto Brasileiro de Geografia e Estatística, IBGE) |
|  | Bolsa Familia Expenditure (per capita) | Municipality | 2013-2017 (yearly average) | Ministry of Social Development and Fight against Hunger (Ministério do Desenvolvimento Social e Combate à Fome, MDS) |
|  | % of extreme poverty among families covered by the cash transfer programme | Municipality | 2013-2017 (yearly average) | Ministry of Social Development and Fight against Hunger (Ministério do Desenvolvimento Social e Combate à Fome, MDS) |
| Education | % 18 years of age or older with a high school degree | Municipality | 2010 | Census, The Brazilian Institute of Geography and Statistics (Instituto Brasileiro de Geografia e Estatística, IBGE) |
| Health and development | Human Development Index (HDI) | Municipality | 2010 | Census, The Brazilian Institute of Geography and Statistics (Instituto Brasileiro de Geografia e Estatística, IBGE) |
| Criminal gang activity | Drug trafficking | Municipality | 2012-2017 (yearly average) | Criminal Statistics (Estatísticas Indicadores Criminais), State Secretary for Public Security (Secretaria de Segurança Pública) |
| COVID-19 | Deaths caused by COVID-19 | Municipality | March 2020- Dec 2021 (monthly average) | [OpenDataSUS](https://opendatasus.saude.gov.br/dataset), Severe Acute Respiratory Syndrome Database - including COVID-19 data (Banco de Dados de Síndrome Respiratória Aguda Grave - incluindo dados da COVID-19). Ministry of Health (Ministério da Saúde) |
|  | Google mobility for workplaces (Locais de Trabalho) during COVID-19 | Municipality | Feb 2020- Dec 2021 (monthly average) | [Painel Google Mobility](https://app.powerbi.com/view?r=eyJrIjoiOGYxODhlYjctYmZkZS00OTE5LTllYmEtNGFkN2FjMzQwNTM4IiwidCI6IjRmZjE0NWRhLThkZWYtNGI3Zi05YTlkLTFiZjRjZDI3MzViYSJ9&pageName=ReportSection6ca03838db8d824dbfe5&pageName=ReportSectiona7fefb4b611090273a96), Estados / Municípios, Governo Federal do Brasil |

***Web-table 8*: Summary of separate synthetic control models for evaluating the effects of Pelotas Pact for Peace, before and during the COVID-19 pandemic**

| **Treatment** | **Date of implementation** | **Post-intervention periods** | **Outcomes** |
| --- | --- | --- | --- |
| **Pelotas Peace Pact (“Pacto”)** | August 1, 2017 | 1. **Full post-intervention:** August 1, 2017, to December 31, 2021 2. **Before pandemic:** August 1, 2017, to February 28, 2020 3. **During pandemic:** March 1, 2020, to December 31, 2021 | - **Violence and crime:** police- and health recorded homicide, crimes against the property, violence against women - **Early risk factor:** school drop-out |
| **Focussed Deterrence Strategy** | May 1, 2018 | 1. **Full post-intervention:** May 1, 2018, to December 31, 2021 2. **Before pandemic:** May 1, 2018, to February 28, 2020 3. **During pandemic:** March 1, 2020, to December 31, 2021 | - **Violence and crime:** police- and health recorded homicide |

***Web-table 9*: Outcome counts before and after the Pelotas Pact for Peace and Focussed Deterrence in Pelotas and 22 control municipalities in Rio Grande do Sul**

|  | | **Total counts^b^** | **Average counts, Mean (SD)^a^** | | | |
| --- | --- | --- | --- | --- | --- | --- |
|  | |  | **Pelotas Pact for Peace, August 2017** | | **Focussed Deterrence, May 2018** | |
|  |  |  | **Pre-intervention** | **Post-intervention** | **Pre-intervention** | **Post-intervention** |
| **Pelotas** | | | | | | |
|  | Police-recorded homicide | 704 | 6·81 (3·59) | 4·68 (3·42) | 7·2 (3·72) | 3·57 (2·08) |
|  | Health-recorded homicide | 744 | 7·37 (3·44) | 6·1 (4) | 7·75 (3·82) | 4·84 (2·37) |
|  | Robbery | 28160 | 269·49 (65·94) | 190·64 (91·31) | .. | .. |
|  | Theft | 38665 | 368·85 (62·81) | 263·25 (53·72) | .. | .. |
|  | Vehicle theft & robbery | 6839 | 70·87 (21·9) | 39·45 (18·83) | .. | .. |
|  | Assault against women | 7475 | 796·4 (90·95) | 698·6 (63·98) | .. | .. |
|  | School dropout | 7786 | 1034·67 (182·94) | 936·4 (168·66) | .. | .. |
| **Donor pool (control municipalities, n=22)** | | | | | | |
|  | Police-recorded homicide | 15049 | 6·61 (11·78) | 4·55 (7·52) | 6·63 (11·77) | 4·09 (6·21) |
|  | Health-recorded homicide | 14958 | 6·91 (12·49) | 5·29 (8·68) | 6·94 (12·48) | 4·76 (7·18) |
|  | Robbery | 500130 | 201·07 (468·27) | 174·74 (432·49) | .. | .. |
|  | Theft | 737117 | 314·41 (557·12) | 234·71 (408·19) | .. | .. |
|  | Vehicle theft & robbery | 209007 | 92·44 (195·9) | 62·4 (137·53) | .. | .. |
|  | Assault against women | 118986 | 603·75 (731·46) | 477·95 (645·72) | .. | .. |
|  | School dropout | 173925 | 895·58 (968·52) | 1043·79 (1170·61) | .. | .. |

^a^Average counts for one municipality are based on yearly data for assault against women and school dropout, and monthly data for all other outcomes.

^b^Total counts based on available time period (variable by outcome, see web-appendix, p 4) for all municipalities.

***Web-table 10*: Average rates of outcomes before and after the Pelotas Pact for Peace and Focussed Deterrence in Pelotas and 22 control municipalities in Rio Grande do Sul**

|  | | **Average rates per 100,000, Mean (SD)^a^** | | | | |
| --- | --- | --- | --- | --- | --- | --- |
|  | | **Available time period^b^** | **Pelotas Pact for Peace, August 2017** | | **Focussed Deterrence, May 2018** | |
|  |  |  | **Pre-intervention** | **Post-intervention** | **Pre-intervention** | **Post-intervention** |
| **Pelotas** | | | | | | |
|  | Police-recorded homicide | 1·73 (1·08) | 2·01 (1·05) | 1·37 (1) | 2·12 (1·09) | 1·04 (0·61) |
|  | Health-recorded homicide | 2·03 (1·09) | 2·18 (1·01) | 1·78 (1·17) | 2·28 (1·12) | 1·41 (0·69) |
|  | Robbery | 69·01 (25·69) | 79·53 (19·21) | 55·71 (26·81) | .. | .. |
|  | Theft | 94·8 (23·69) | 108·97 (18·79) | 76·87 (15·81) | .. | .. |
|  | Vehicle theft & robbery | 16·78 (7·65) | 20·93 (6·47) | 11·53 (5·53) | .. | .. |
|  | Assault against women | 429·41 (51·87) | 457·5 (52·25) | 401·32 (36·75) | .. | .. |
|  | School dropout | 1430·83 (299·68) | 1415·65 (302·71) | 1439·93 (333·27) | .. | .. |
| **Donor pool (control municipalities, n=22)** | | | | | | |
|  | Police-recorded homicide | 2·09 (1·79) | 2·37 (1·89) | 1·73 (1·58) | 2·38 (1·93) | 1·58 (1·37) |
|  | Health-recorded homicide | 2·3 (1·79) | 2·47 (1·85) | 2 (1·64) | 2·48 (1·88) | 1·85 (1·44) |
|  | Robbery | 54·82 (40·45) | 60·38 (41·14) | 47·79 (38·46) | .. | .. |
|  | Theft | 104·91 (34·59) | 119·26 (32·22) | 86·75 (28·41) | .. | .. |
|  | Vehicle theft & robbery | 24·33 (17·43) | 28·66 (18·29) | 18·85 (14·54) | .. | .. |
|  | Assault against women | 476·9 (115·31) | 536·62 (106·48) | 417·17 (90·43) | .. | .. |
|  | School dropout | 2265·26 (1158·71) | 1818·67 (606·36) | 2533·21 (1320·26) | .. | .. |

^a^Average rates for one municipality are based on yearly data for assault against women and school dropout, and monthly data for all other outcomes. For assault against women rates are derived using yearly female population estimates and for school dropout rates are derived using the total of pupils initially enrolled in schools by municipality.

^b^Available time period is variable (see web-appendix, p 4).

***Web-table 11*: Weights for synthetic controls for evaluating the effects of Pelotas Pact for Peace**

|  | **Pelotas Pact for Peace, August 2017** | | | | | | | **Focussed Deterrence, May 2018** | |
| --- | --- | --- | --- | --- | --- | --- | --- | --- | --- |
| **Municipality** | **Homicide** | | **Property crime** | | | **Yearly outcomes** | | **Homicide** | |
|  | **Police-recorded** | **Health-recorded** | **Robbery** | **Theft** | **Motor vehicle theft & robbery** | **Assault against women** | **School dropout** | **Police-recorded** | **Health-recorded** |
| Alvorada | 0 | 0 | 0 | 0 | 0 | 0 | 0 | 0·05 | 0 |
| Bage | 0·11 | 0 | 0 | 0 | 0 | 0 | 0·54 | 0·09 | 0·07 |
| Bento Goncalves | 0·02 | 0 | 0 | 0 | 0 | 0 | 0·29 | 0·16 | 0·14 |
| Cachoeira do Sul | 0 | 0 | 0 | 0·11 | 0 | 0 | 0·09 | 0 | 0 |
| Cachoeirinha | 0·2 | 0·12 | 0 | 0 | 0 | 0 | 0 | 0·15 | 0 |
| Caxias do Sul | 0·28 | 0·18 | 0 | 0 | 0 | 0·42 | 0 | 0·24 | 0·16 |
| Erechim | 0 | 0 | 0 | 0 | 0·05 | 0 | 0 | 0 | 0 |
| Esteio | 0 | 0·15 | 0·09 | 0·11 | 0 | 0 | 0 | 0 | 0 |
| Gravatai | 0 | 0·09 | 0 | 0·4 | 0 | 0 | 0 | 0 | 0·07 |
| Guaiba | 0 | 0·07 | 0 | 0 | 0 | 0 | 0·02 | 0 | 0·11 |
| Ijui | 0·2 | 0 | 0 | 0 | 0 | 0·26 | 0 | 0·17 | 0 |
| Novo Hamburgo | 0 | 0 | 0·24 | 0 | 0·05 | 0·01 | 0 | 0 | 0 |
| Passo Fundo | 0 | 0·02 | 0·09 | 0 | 0 | 0 | 0 | 0 | 0·11 |
| Porto Alegre | 0·19 | 0 | 0·15 | 0 | 0·1 | 0 | 0·04 | 0·14 | 0 |
| Rio Grande | 0 | 0 | 0·13 | 0 | 0·61 | 0 | 0 | 0 | 0·12 |
| Santa Cruz do Sul | 0 | 0 | 0·21 | 0 | 0 | 0 | 0·02 | 0 | 0 |
| Santa Maria | 0 | 0·27 | 0 | 0·19 | 0 | 0 | 0 | 0 | 0 |
| Sao Leopoldo | 0 | 0·03 | 0 | 0 | 0 | 0 | 0 | 0 | 0 |
| Sapiranga | 0 | 0·08 | 0 | 0 | 0·03 | 0 | 0 | 0 | 0·12 |
| Sapucaia do Sul | 0 | 0 | 0·09 | 0 | 0 | 0 | 0 | 0 | 0 |
| Uruguaiana | 0 | 0 | 0 | 0 | 0·15 | 0·31 | 0 | 0 | 0 |
| Viamao | 0 | 0 | 0 | 0·2 | 0 | 0 | 0 | 0 | 0·09 |

Synthetic control weights for each synthetic control (represented by each separate column) add up to 1, small deviations of 0.01 reflect rounding error.

***Web-table 12*: Average covariate balance before Pelotas Pact for Peace was introduced for Pelotas and the synthetic controls, police- and health-recorded homicide**

|  |  | **Pelotas Pact for Peace, August 2017** | | | | | | | | **Focussed Deterrence, May 2018** | | | | | | | | |
| --- | --- | --- | --- | --- | --- | --- | --- | --- | --- | --- | --- | --- | --- | --- | --- | --- | --- | --- |
|  |  | **Police-recorded homicide** | | | | **Health-recorded homicide** | | | | **Police-recorded homicide** | | | | **Health-recorded homicide** | | | | |
|  |  | **Pelotas** | **Unweighted control** | **Synthetic (weighted) control** | **Variable weights** | **Pelotas** | **Unweighted control** | **Synthetic (weighted) control** | **Variable weights** | **Pelotas** | **Unweighted control** | **Synthetic (weighted) control** | **Variable weights** | **Pelotas** | **Unweighted control** | **Synthetic (weighted) control** | **Variable weights** |  |
| **Population sociodemographic,** % | | | | | | | | | | | | | | | | | | |
|  | Sex: Male | 46·97 | 48·32 | 48·12 | 0 | 46·97 | 48·32 | 48·32 | 0 | 46·97 | 48·32 | 48·31 | 0 | 46·97 | 48·32 | 48·61 | 0 |  |
|  | Age: 5-24years | 30·77 | 32·12 | 30·8 | 0·85 | 30·77 | 32·12 | 32·07 | 0 | 30·77 | 32·12 | 30·84 | 0·72 | 30·77 | 32·12 | 32·22 | 0 |  |
|  | Age: 25-34years | 15·65 | 16·68 | 17·23 | 0 | 15·65 | 16·68 | 17·11 | 0 | 15·65 | 16·68 | 17·38 | 0 | 15·65 | 16·68 | 17·05 | 0 |  |
|  | Age: 35-54years | 27·11 | 27·59 | 27·88 | 0 | 27·11 | 27·59 | 27·75 | 0·01 | 27·11 | 27·59 | 27·99 | 0 | 27·11 | 27·59 | 27·65 | 0 |  |
|  | Age: 55years+ | 20·81 | 17·31 | 18·09 | 0 | 20·81 | 17·31 | 16·81 | 0 | 20·81 | 17·31 | 17·84 | 0 | 20·81 | 17·31 | 16·78 | 0 |  |
|  | Race: White | 80·25 | 82·78 | 82·18 | 0·02 | 80·25 | 82·78 | 84·3 | 0 | 80·25 | 82·78 | 82·49 | 0·04 | 80·25 | 82·78 | 82·27 | 0 |  |
|  | Race: Black | 10·68 | 5·9 | 5·5 | 0 | 10·68 | 5·9 | 5·06 | 0 | 10·68 | 5·9 | 5·23 | 0 | 10·68 | 5·9 | 5·51 | 0 |  |
|  | Race: Other races | 9·07 | 11·32 | 12·31 | 0 | 9·07 | 11·32 | 10·65 | 0·01 | 9·07 | 11·32 | 12·28 | 0 | 9·07 | 11·32 | 12·21 | 0 |  |
| **Economic** | | | | | | | | | | | | | | | | | | |
|  | Living in poverty, % | 7·56 | 5·42 | 4·2 | 0 | 7·56 | 5·42 | 4·37 | 0 | 7·56 | 5·42 | 3·94 | 0 | 7·56 | 5·42 | 4·56 | 0 |  |
|  | Bolsa expenditure per capita | 32·93 | 43·1 | 39·92 | 0 | 32·93 | 43·1 | 36·24 | 0 | 31·87 | 42·42 | 36·15 | 0 | 31·87 | 42·42 | 37·19 | 0·01 |  |
|  | Bolsa coverage for extreme poverty, % | 18·47 | 22·48 | 25·58 | 0 | 18·47 | 22·48 | 21·7 | 0 | 18·25 | 22·52 | 23·53 | 0 | 18·25 | 22·52 | 18·6 | 0 |  |
| **Education** | | | | | | | | | | | | | | | | | | |
|  | High school educated, % | 40·96 | 41·07 | 46·1 | 0 | 40·96 | 41·07 | 44·1 | 0 | 40·96 | 41·07 | 44·84 | 0 | 40·96 | 41·07 | 40·11 | 0·02 |  |
| **Health and development** | | | | | | | | | | | | | | | | | | |
|  | Human Development Index | 0·74 | 0·75 | 0·78 | 0 | 0·74 | 0·75 | 0·76 | 0 | 0·74 | 0·75 | 0·77 | 0 | 0·74 | 0·75 | 0·75 | 0·02 |  |
| **Criminal gang activity** | | | | | | | | | | | | | | | | | | |
|  | Drug trafficking, average monthly rate per 100,000 | 5·82 | 7·43 | 7·17 | 0·01 | 5·82 | 7·43 | 6·15 | 0·11 | 5·95 | 7·51 | 7·05 | 0·02 | 5·95 | 7·51 | 6·52 | 0·01 |  |
| **Homicide, average monthly rate per 100,000** | | | | | | | | | | | | | | | | | | |
|  | 2012 | 1·46 | 2·02 | 1·76 | 0 | 1·53 | 2·17 | 2 | 0·01 | 1·46 | 2·02 | 1·85 | 0 | 1·53 | 2·17 | 1·87 | 0·01 |  |
|  | 2013 | 1·53 | 1·98 | 1·73 | 0·02 | 1·8 | 2·11 | 1·84 | 0·21 | 1·53 | 1·98 | 1·78 | 0·01 | 1·8 | 2·11 | 2·01 | 0·01 |  |
|  | 2014 | 1·82 | 2·52 | 2 | 0·01 | 2 | 2·56 | 2·21 | 0·01 | 1·82 | 2·52 | 2·14 | 0 | 2 | 2·56 | 2·21 | 0 |  |
|  | 2015 | 2·78 | 2·44 | 2·06 | 0·02 | 2·83 | 2·51 | 2·31 | 0·08 | 2·78 | 2·44 | 2·12 | 0·07 | 2·83 | 2·51 | 2·33 | 0·01 |  |
|  | 2016 | 1·89 | 2·55 | 2·11 | 0·03 | 2·25 | 2·67 | 2·41 | 0·06 | 1·89 | 2·55 | 2·17 | 0·12 | 2·25 | 2·67 | 2·53 | 0 |  |
|  | 2017^a^ | 2·98 | 2·93 | 2·6 | 0·04 | 2·98 | 3·09 | 2·99 | 0·51 | 2·91 | 2·82 | 2·54 | 0·02 | 2·88 | 2·94 | 2·89 | 0·91 |  |
|  | 2018^b^ | .. | .. | .. | .. | .. | .. | .. | .. | 3·15 | 2·17 | 2·48 | 0 | 3·51 | 2·32 | 2·51 | 0·01 |  |

^a^Average rate from January 1, 2017, to July 31, 2017, since the Pelotas Pact for Peace was introduced in August 2017.

^b^Average rate from January 1, 2018, to July 31, 2017, since the Pelotas Pact for Peace was introduced in May 2018.

***Web-table 13*: Average covariate balance before Pelotas Pact for Peace was introduced for Pelotas and the synthetic controls, property crime**

|  |  | **Robbery** | | | | **Theft** | | | | **Vehicle theft & robbery** | | | |
| --- | --- | --- | --- | --- | --- | --- | --- | --- | --- | --- | --- | --- | --- |
|  |  | **Pelotas** | **Unweighted control** | **Synthetic (weighted) control** | **Variable weights** | **Pelotas** | **Unweighted control** | **Synthetic (weighted) control** | **Variable weights** | **Pelotas** | **Unweighted control** | **Synthetic (weighted) control** | **Variable weights** |
| **Population sociodemographic,** % | | | | | |  |  |  |  |  |  |  |  |
|  | Sex: Male | 46·97 | 48·32 | 47·97 | 0 | 46·97 | 48·32 | 48·39 | 0 | 46·97 | 48·32 | 48·11 | 0 |
|  | Age: 5-24years | 30·77 | 32·12 | 31·2 | 0 | 30·77 | 32·12 | 32·57 | 0 | 30·77 | 32·12 | 32 | 0 |
|  | Age: 25-34years | 15·65 | 16·68 | 16·89 | 0 | 15·65 | 16·68 | 16·39 | 0 | 15·65 | 16·68 | 16·23 | 0 |
|  | Age: 35-54years | 27·11 | 27·59 | 27·93 | 0 | 27·11 | 27·59 | 26·97 | 0 | 27·11 | 27·59 | 26·86 | 0 |
|  | Age: 55years+ | 20·81 | 17·31 | 17·94 | 0 | 20·81 | 17·31 | 17·65 | 0 | 20·81 | 17·31 | 18·55 | 0 |
|  | Race: White | 80·25 | 82·78 | 85·13 | 0 | 80·25 | 82·78 | 82·58 | 0 | 80·25 | 82·78 | 80·17 | 0 |
|  | Race: Black | 10·68 | 5·9 | 5·79 | 0 | 10·68 | 5·9 | 7·57 | 0 | 10·68 | 5·9 | 7·26 | 0 |
|  | Race: Other races | 9·07 | 11·32 | 9·08 | 0·85 | 9·07 | 11·32 | 9·85 | 0·01 | 9·07 | 11·32 | 12·55 | 0 |
| **Economic** | | | | | | | | | | | | | |
|  | Living in poverty, % | 7·56 | 5·42 | 4·37 | 0 | 7·56 | 5·42 | 6·06 | 0 | 7·56 | 5·42 | 7·03 | 0·01 |
|  | Bolsa expenditure per capita | 32·93 | 43·1 | 38·54 | 0 | 32·93 | 43·1 | 50·56 | 0 | 32·93 | 43·1 | 41·99 | 0 |
|  | Bolsa coverage for extreme poverty, % | 18·47 | 22·48 | 20·86 | 0 | 18·47 | 22·48 | 32·78 | 0 | 18·47 | 22·48 | 19·55 | 0 |
| **Education** | | | | | | | | | | | | | |
|  | High school educated, % | 40·96 | 41·07 | 43·89 | 0 | 40·96 | 41·07 | 40·78 | 0·26 | 40·96 | 41·07 | 41·53 | 0 |
| **Health and development** | | | | | | | | | | | | | |
|  | Human Development Index | 0·74 | 0·75 | 0·76 | 0 | 0·74 | 0·75 | 0·74 | 0·04 | 0·74 | 0·75 | 0·75 | 0 |
| **Criminal gang activity** | | | | | | | | | | | | | |
|  | Drug trafficking, average monthly rate per 100,000 | 5·82 | 7·43 | 8·96 | 0 | 5·82 | 7·43 | 6·96 | 0 | 5·82 | 7·43 | 9·8 | 0 |
| **Property crime, average monthly rate per 100,000** | | | | | | | | | | | | | |
|  | 2012 | 60·6 | 41·61 | 58·7 | 0 | 111·12 | 125·69 | 111·92 | 0 | 14·68 | 23·7 | 16·15 | 0·02 |
|  | 2013 | 69·81 | 46·88 | 64·3 | 0 | 131·59 | 124·43 | 122·3 | 0·01 | 22·69 | 24·16 | 19·8 | 0·01 |
|  | 2014 | 69·91 | 54·97 | 71·87 | 0 | 119·51 | 127·8 | 119·71 | 0·65 | 25·08 | 28·35 | 22·26 | 0 |
|  | 2015 | 89·06 | 71·57 | 93·6 | 0 | 93·63 | 115·82 | 103·54 | 0 | 26·02 | 33·24 | 25·01 | 0·28 |
|  | 2016 | 91·97 | 75·82 | 93·82 | 0·11 | 97·43 | 112·47 | 103·37 | 0 | 16·76 | 32·54 | 21·6 | 0 |
|  | 2017^a^ | 107·5 | 79·28 | 99·5 | 0·02 | 94·55 | 102·3 | 92·8 | 0·04 | 19·91 | 30·87 | 20·3 | 0·68 |

^a^Average rate from January 1, 2017, to July 31, 2017, since the Pelotas Pact for Peace was introduced in August 2017.

***Web-table 14*: Effects of Pelotas Peace Pact on health-recorded homicide before and during the COVID-19 pandemic, compared with synthetic controls**

|  |  | **Full post-intervention period** | | | **Before the COVID-19 pandemic** | | | **During the COVID-19 pandemic** | | |
| --- | --- | --- | --- | --- | --- | --- | --- | --- | --- | --- |
|  |  | **Synthetic control** | **Pelotas** | **Difference, absolute (relative)** | **Synthetic control** | **Pelotas** | **Difference, absolute (relative)** | **Synthetic control** | **Pelotas** | **Difference, absolute (relative)** |
| **Pelotas Pact for Peace, August 2017** | | | | | | | | | | |
|  | Health-recorded homicide | 1·76 | 1·78 | 0·03 (2%) | 1·86 | 2·06 | 0·2 (11%) | 1·44 | 0·93 | -0·51 (-35%) |
| **Focussed Deterrence, May 2018** | | | | | | | | | | |
|  | Health-recorded homicide | 1·94 | 1·41 | -0·52 (-27%) | 2·02 | 1·63 | -0·39 (-19%) | 1·74 | 0·93 | -0·81 (-46%) |

Monthly rates span from January 1, 2012, until December 31, 2020. Before the COVID-19 pandemic spans up until February 28, 2020, and during the pandemic spans from March 1, 2020, onwards. The relative difference between Pelotas and the synthetic control is represented by the total percentage difference while the absolute difference is represented by the average monthly difference.

***Web-table 15*: Placebo tests and robust t-tests estimating the significance of the effects of Pelotas Peace Pact,** **before and during the COVID-19 pandemic**

|  | | **Placebo test** | | | **Robust t-test** | | |
| --- | --- | --- | --- | --- | --- | --- | --- |
|  | | **Quasi *p* value** | | | **Average monthly difference (95% CI)** | | |
|  |  | **Full post-intervention period** | **Before the COVID-19 pandemic** | **During the COVID-19 pandemic** | **Full post-intervention period** | **Before the COVID-19 pandemic** | **During the COVID-19 pandemic** |
| **Pelotas Pact for Peace, August 2017** | | | | | | | |
|  | Police-recorded homicide | 5/23=0·22 | 16/23=0·70 | 9/23=0·39 | -0·15 (-2·4, 2·1) | 0·16 (-2·23, 2·55) | -0·59 (-2·64, 1·46) |
|  | Health-recorded homicide | 4/25=0·16 | 9/25=0·36 | 6/25=0·24 | 0·06 (-1·34, 1·46) | 0·22 (-1·02, 1·46) | -0·42 (-2·32, 1·47) |
|  | Robbery | 19/23=0·83 | 14/23=0·61 | 5/23=0·22 | -5·24 (-35·51, 25·03) | 1·14 (-31·35, 33·62) | -14·23 (-41·46, 13) |
|  | Theft | 7/23=0·30 | 23/23=1.00 | 13/23=0·57 | -0·19 (-20·89, 20·5) | 5 ·00 (-15·61, 25·61) | -7·51 (-28·35, 13·33) |
|  | Vehicle theft & robbery | 7/23=0·30 | 23/23=1.00 | 13/23=0·57 | -1·97 (-17·14, 13·2) | -1·15 (-15·63, 13·33) | -3·13 (-19·27, 13·01) |
|  | Assault against women^†^ | 6/23=0·26 |  |  | .. | .. | .. |
|  | School dropout^†^ | 9/23=0·39 |  |  | .. | .. | .. |
| **Focussed Deterrence, May 2018** | | | | | | | |
|  | Police-recorded homicide | 5/23=0·22 | 18/23=0·78 | 3/23=0·13 | -0·63 (-2·21, 0·94) | -0·43 (-2·21, 1·35) | -0·83 (-2·35, 0·7) |
|  | Health-recorded homicide | 5/23=0·22 | 17/23=0·74 | 5/23=0·22 | -0·41 (-2·03, 1·22) | -0·3 (-2·26, 1·65) | -0·63 (-1·55, 0·3) |

Monthly police-recorded crime span from January 1, 2012, until December 31, 2021, while heath-recorded homicide span from January 1, 2012, until December 31, 2020. Before the COVID-19 pandemic spans up until February 28, 2020, and during the pandemic spans from March 1, 2020, onwards. Quasi *p* values are computed from placebo tests,^2^ while the robust-tests derive bias corrected average (absolute) monthly difference and their corresponding 95% confidence intervals (CI ) using cross-fitting.^10^ See Table 1 in the manuscript for corresponding crude estimates.

^†^Yearly rates which are derived using yearly female population estimates and total pupils initially enrolled in schools by municipality.

* *p* < ·05; ** *p* < ·01; *** *p* < ·001

***Web-table 16*: Average covariate balance before Pelotas Pact for Peace was introduced for Pelotas and the synthetic controls, yearly outcomes**

|  |  | **Assaults against women** | | | | **School dropout** | | | |
| --- | --- | --- | --- | --- | --- | --- | --- | --- | --- |
|  |  | **Pelotas** | **Unweighted control** | **Synthetic (weighted) control** | **Variable weights** | **Pelotas** | **Unweighted control** | **Synthetic (weighted) control** | **Variable weights** |
| **Population sociodemographic,** % | | | | | |  |  |  |  |
|  | Sex: Male | 46·97 | 48·32 | 48·72 | 0·00 | 46·97 | 48·32 | 48·15 | 0 |
|  | Age: 5-24years | 30·77 | 32·12 | 32·16 | 0·00 | 30·77 | 32·12 | 30·98 | 0 |
|  | Age: 25-34years | 15·65 | 16·68 | 16·96 | 0·00 | 15·65 | 16·68 | 15·99 | 0 |
|  | Age: 35-54years | 27·11 | 27·59 | 27·53 | 0·00 | 27·11 | 27·59 | 27·67 | 0 |
|  | Age: 55years+ | 20·81 | 17·31 | 16·83 | 0·00 | 20·81 | 17·31 | 19·54 | 0 |
|  | Race: White | 80·25 | 82·78 | 80·61 | 0·00 | 80·25 | 82·78 | 80·25 | 0·03 |
|  | Race: Black | 10·68 | 5·90 | 3·36 | 0·00 | 10·68 | 5·9 | 7·07 | 0 |
|  | Race: Other races | 9·07 | 11·32 | 16·03 | 0·00 | 9·07 | 11·32 | 12·68 | 0 |
| **Economic** | | | | | | |  |  |  |
|  | Living in poverty, % | 7·56 | 5·42 | 6·07 | 0·00 | 7·56 | 5·42 | 6·41 | 0 |
|  | Bolsa expenditure per capita | 34·78 | 44·28 | 44·33 | 0·00 | 31·11 | 42·7 | 63·68 | 0 |
|  | Bolsa coverage for extreme poverty, % | 18·88 | 22·41 | 23·66 | 0·00 | 18·88 | 22·41 | 27·7 | 0 |
| **Education** | | | | | | |  |  |  |
|  | High school educated, % | 40·96 | 41·07 | 42·61 | 0·01 | 40·96 | 41·07 | 40·52 | 0 |
| **Health and development** | | | | | | |  |  |  |
|  | Human Development Index | 0·74 | 0·75 | 0·77 | 0·00 | 0·74 | 0·75 | 0·75 | 0 |
| **Criminal gang activity** | | | | | | |  |  |  |
|  | Drug trafficking, average monthly rate per 100,000 | 66·33 | 88·86 | 67·59 | 0·00 | 77·13 | 88·71 | 77·13 | 0·01 |
| **Average yearly rate per 100,000^a^** | | | | | | |  |  |  |
|  | 2012 | 516·44 | 576·81 | 517·99 | 0·63 | .. | .. | .. | .. |
|  | 2013 | 474·50 | 564·63 | 467·42 | 0·18 | .. | .. | .. | .. |
|  | 2014 | 491·16 | 546·62 | 483·71 | 0·06 | 1762·4 | 2059·34 | 1762·4 | 0·15 |
|  | 2015 | 405·57 | 518·44 | 420·91 | 0·06 | 1280·47 | 1838·37 | 1280·47 | 0·15 |
|  | 2016 | 399·82 | 476·62 | 407·53 | 0·07 | 1204·09 | 1558·31 | 1204·09 | 0·15 |

^a^For assault against women rates are derived using yearly female population estimates and for school dropout rates are derived using the total of pupils initially enrolled in schools by municipality.

***Web-table 17*: Sensitivity analysis for the effects of Pelotas Peace Pact on monthly violence and crime, controlling for monthly COVID-19 measures (COVID-19 related deaths and google mobility)**

|  |  | **Full post-intervention period** | | | **Before the COVID-19 pandemic** | | | **During the COVID-19 pandemic** | | |
| --- | --- | --- | --- | --- | --- | --- | --- | --- | --- | --- |
|  |  | **Synthetic control** | **Pelotas** | **Difference, absolute (relative)** | **Synthetic control** | **Pelotas** | **Difference, absolute (relative)** | **Synthetic control** | **Pelotas** | **Difference, absolute (relative)** |
| **Pelotas Pact for Peace, August 2017** | | | | | | | | | | |
|  | Homicide | 1·49 | 1·37 | -0·13 (-8%) | 1·62 | 1·86 | 0·24 (15%) | 1·31 | 0·67 | -0·64 (-48%) |
|  | Robbery | 59·83 | 55·71 | -4·12 (-7%) | 73·26 | 74·63 | 1·38 (2%) | 40·92 | 29·06 | -11·86 (-29%) |
|  | Theft | 78·36 | 76·87 | -1·49 (-2%) | 84·44 | 88·11 | 3·66 (4%) | 69·78 | 61·04 | -8·75 (-13%) |
|  | Vehicle theft & robbery | 13·06 | 11·53 | -1·53 (-12%) | 15·97 | 15·3 | -0·67 (-4%) | 8·95 | 6·22 | -2·74 (-31%) |
| **Focussed Deterrence, May 2018** | | | | | | | | | | |
|  | Homicide | 1·68 | 1·04 | -0·63 (-38%) | 1·85 | 1·41 | -0·44 (-24%) | 1·5 | 0·67 | -0·83 (-55%) |

Before the COVID-19 pandemic spans up until February 28, 2020, and during the pandemic spans from March 1, 2020, onwards. The relative difference between Pelotas and the synthetic control is represented by the total percentage difference while the absolute difference is represented by the average yearly difference.

***Web-table 18*: Sensitivity analysis for the effects of Pelotas Peace Pact on monthly violence and crime, pre-filtering the monthly outcome series**

|  |  | **Full post-intervention period** | | | **Before the COVID-19 pandemic** | | | **During the COVID-19 pandemic** | | |
| --- | --- | --- | --- | --- | --- | --- | --- | --- | --- | --- |
|  |  | **Synthetic control** | **Pelotas** | **Difference, absolute (relative)** | **Synthetic control** | **Pelotas** | **Difference, absolute (relative)** | **Synthetic control** | **Pelotas** | **Difference, absolute (relative)** |
| **Pelotas Pact for Peace, August 2017** | | | | | | | | | | |
|  | Homicide | 1·57 | 1·37 | -0·2 (-13%) | 1·74 | 1·86 | 0·12 (7%) | 1·34 | 0·67 | -0·66 (-50%) |
|  | Robbery | 60·3 | 55·71 | -4·59 (-8%) | 72·46 | 74·63 | 2·17 (3%) | 43·17 | 29·06 | -14·12 (-33%) |
|  | Theft | 80·67 | 76·87 | -3·79 (-5%) | 86·13 | 88·11 | 1·98 (2%) | 72·97 | 61·04 | -11·93 (-16%) |
|  | Vehicle theft & robbery | 12·77 | 11·53 | -1·24 (-10%) | 16·11 | 15·3 | -0·81 (-5%) | 8·06 | 6·22 | -1·84 (-23%) |
| **Focussed Deterrence, May 2018** | | | | | | | | | | |
|  | Homicide | 1·6 | 1·04 | -0·56 (-35%) | 1·64 | 1·41 | -0·24 (-14%) | 1·56 | 0·67 | -0·88 (-57%) |

Before the COVID-19 pandemic spans up until February 28, 2020, and during the pandemic spans from March 1, 2020, onwards. The relative difference between Pelotas and the synthetic control is represented by the total percentage difference while the absolute difference is represented by the average yearly difference.

***Web-table 19*: Sensitivity analysis for the effects of Pelotas Peace Pact on crime, violence, and school dropout, excluding Porto Alegre from the donor pool**

|  | | | **Full post-intervention period** | | | **Before the COVID-19 pandemic** | | | **During the COVID-19 pandemic** | | |
| --- | --- | --- | --- | --- | --- | --- | --- | --- | --- | --- | --- |
|  |  |  | **Synthetic control** | **Pelotas** | **Difference, absolute (relative)** | **Synthetic control** | **Pelotas** | **Difference, absolute (relative)** | **Synthetic control** | **Pelotas** | **Difference, absolute (relative)** |
| **Monthly police-recorded crime rates per 100,000 residents** | | | | | | | | | | | |
|  | **Pelotas Peace Pact, August 2017** | | | | | | | | | | |
|  |  | Homicide | 1·57 | 1·37 | -0·2 (-13%) | 1·67 | 1·86 | 0·19 (11%) | 1·42 | 0·67 | -0·75 (-53%) |
|  |  | Robbery | 61·9 | 55·71 | -6·19 (-10%) | 75·16 | 74·63 | -0·53 (-1%) | 43·22 | 29·06 | -14·16 (-33%) |
|  |  | Theft | 78·36 | 76·87 | -1·49 (-2%) | 84·44 | 88·11 | 3·66 (4%) | 69·78 | 61·04 | -8·75 (-13%) |
|  |  | Vehicle theft & robbery | 12·68 | 11·53 | -1·15 (-9%) | 15·37 | 15·3 | -0·07 (0%) | 8·88 | 6·22 | -2·66 (-30%) |
|  | **Focussed Deterrence, May 2018** | | | | | | | | | | |
|  |  | Homicide | 1·7 | 1·04 | -0·66 (-39%) | 1·9 | 1·41 | -0·49 (-26%) | 1·5 | 0·67 | -0·82 (-55%) |
| **Yearly rates of assault against women and school drop-out per 100,000**^†^ | | | | | | | | | | | |
|  | **Pelotas Peace Pact, August 2017** | | | | | | | | | | |
|  |  | Assault against women | 417·78 | 401·32 | -16·46 (-4%) | .. | .. | .. | .. | .. | .. |
|  |  | School dropout | 2560·46 | 1439·93 | -1120·53 (-44%) | .. | .. | .. | .. | .. | .. |

Monthly crime rates span from January 1, 2012, until December 31, 2021, while yearly rates of assaults against women span from 2012 until 2021 and school dropout from 2014 until 2021. Before the COVID-19 pandemic spans up until February 28, 2020, and during the pandemic spans from March 1, 2020, onwards. The relative difference between Pelotas and the synthetic control is represented by the total percentage difference while the absolute difference is represented by the average yearly difference.

^†^Rates are derived using yearly female population estimates and total pupils initially enrolled in schools by municipality.

***Web-figure 1:* A schematic logic model of the effects of the Pelotas Peace Pact violence and crime**

***Web-figure 2:* A schematic diagram of the proposed strands and targeted interventions delivered under the Social Prevention Axis**

******

***Web-figure 3:* A schematic illustration of the proposed strands and targeted interventions delivered under the Policing and Justice Axis****

***Web-figure 4:* A schematic illustration of the proposed strands and targeted interventions delivered under Administration, Urbanism, and Technology Axes**

***Web-figure 5:* A** **map of municipalities in Rio Grande do Sul, highlighting Pelotas (treated unit) and a donor pool of 22 potential control units (with population sizes ≥80,000 and without similar city-wide interventions [Canoas, Lajeado])**

***Web-figure 6:* Size of the estimated (placebo) effects of the Pelotas Pact for Peace on police-recorded homicide standardized by pre-intervention fit in all municipalities (ordered from largest to smallest), for the full post-intervention period**

Red represents the true treated municipality of Pelotas.

******

***Web-figure 7:* Size of the estimated (placebo) effects of the Pelotas Pact for Peace on police-recorded homicide standardized by pre-intervention fit in all municipalities (ordered from largest to smallest), before and during the COVID-19 pandemic**

Red represents the true treated municipality of Pelotas.

******

***Web-figure 8:* Size of the estimated (placebo) effects of the Focussed Deterrence Strategy on police-recorded homicide standardized by pre-intervention fit in all municipalities (ordered from largest to smallest), for the full post-intervention period**

Red represents the true treated municipality of Pelotas.

***Web-figure 9:* Size of the estimated (placebo) effects of the Focussed Deterrence Strategy on police-recorded homicide standardized by pre-intervention fit in all municipalities (ordered from largest to smallest), before and during the COVID-19 pandemic**

Red represents the true treated municipality of Pelotas.

******

***Web-figure 10:* Size of the estimated (placebo) effects of the Pelotas Pact for Peace on robbery standardized by pre-intervention fit in all municipalities (ordered from largest to smallest), for the full post-intervention period**

Red represents the true treated municipality of Pelotas.

***Web-figure 11:* Size of the estimated (placebo) effects of the Pelotas Pact for Peace on robbery standardized by pre-intervention fit in all municipalities (ordered from largest to smallest), before and during the COVID-19 pandemic**

Red represents the true treated municipality of Pelotas.

******

***Web-figure 12:* Size of the estimated (placebo) effects of the Pelotas Pact for Peace on theft standardized by pre-intervention fit in all municipalities (ordered from largest to smallest), for the full post-intervention period**

Red represents the true treated municipality of Pelotas.

***Web-figure 13:* Size of the estimated (placebo) effects of the Pelotas Pact for Peace on theft standardized by pre-intervention fit in all municipalities (ordered from largest to smallest), before and during the COVID-19 pandemic**

Red represents the true treated municipality of Pelotas.

******

***Web-figure 14:* Size of the estimated (placebo) effects of the Pelotas Pact for Peace on vehicle theft and robbery standardized by pre-intervention fit in all municipalities (ordered from largest to smallest), for the full post-intervention period**

Red represents the true treated municipality of Pelotas.

***Web-figure 15:* Size of the estimated (placebo) effects of the Pelotas Pact for Peace on vehicle theft and robbery standardized by pre-intervention fit in all municipalities (ordered from largest to smallest), before and during the COVID-19 pandemic**

Red represents the true treated municipality of Pelotas.

***Web-figure 16:* Controlled interrupted time series estimates for the effect of the Pelotas Peace Pact and Focussed Deterrence on police-recorded homicide, compared with synthetic controls, before and during the COVID-19 pandemic.**

The solid vertical line represents the introduction of the intervention of Pelotas Peace Pact in August 2017 (*top panels*) and Focussed Deterrence in May 2018 (*bottom panels*); and the dashed vertical line represents the beginning of the COVID-19 period in South Brazil. Solid straight lines represent smoothed trends for homicide rates while shaded ribbons represent 95% confidence intervals around smoothed estimates.

***Web-figure 17:* Controlled interrupted time series estimates for the effect of the Pelotas Peace Pact on property crime, compared with synthetic controls, before and during the COVID-19 pandemic**

The solid vertical line represents the introduction of the intervention of Pelotas Peace Pact in August 2017 and the dashed vertical line represents the beginning of the COVID-19 period in South Brazil. Solid straight lines represent smoothed trends for crime rates while shaded ribbons represent 95% confidence intervals around smoothed estimates.

**

**Web-references**

1 Bonander C, Degli Esposti M. Synthetic Control Methods for the Evaluation of Single-Unit Interventions in Epidemiology: A Tutorial. *Am J Epidemiol* 2021. DOI:10.1093/aje/kwab211.

2 Abadie A, Diamond A, Hainmueller J. Synthetic control methods for comparative case studies: estimating the effect of California’s Tobacco Control Program. *J Am Stat Assoc* 2010; **105**: 493–505.

3 Chernozhukov V, Wuthrich K, Zhu Y. Practical and robust t-test based inference for synthetic control and related methods. *ArXiv Prepr* 2020. https://drive.google.com/file/d/1DS6u6WCLh-Ioea5DOSaszVL1m-MdFHze/view.

4 Ministério da Saúde, Governo Federal do Brasil. DATASUS: Morbidade Hospitalar do SUS por Causas Externas - por local de residência - Rio Grande do Sul. http://tabnet.datasus.gov.br/cgi/deftohtm.exe?sih/cnv/frrs.def (accessed Sept 14, 2021).

5 Borges D, Rojido E, Cano I. Avaliação de Impacto do Pacto Pelotas pela Paz. Universidade do Estado do Rio de Janeiro, 2020 http://www.lav.uerj.br/docs/rel/2020/Avalia%C3%A7%C3%A3o%20de%20Impacto%20do%20Pacto%20Pelotas.pdf (accessed July 1, 2021).

6 Rio Grande do Sul. Secretaria de Segurança Pública. Estatísticas. 2021. https://www.ssp.rs.gov.br/estatisticas (accessed Nov 16, 2021).

7 Ministério da Saúde. Morbidade Hospitalar do SUS por Causas Externas - por local de residência - Rio Grande do Sul. http://tabnet.datasus.gov.br/cgi/deftohtm.exe?sih/cnv/frrs.def (accessed Sept 14, 2021).

8 Ministério da Saúde. Governo Federal do Brasil. DATASUS. https://datasus.saude.gov.br/ (accessed Nov 16, 2021).

9 Secretaria da Educação. Secr. Educ. https://educacao.rs.gov.br/inicial (accessed Nov 16, 2021).

10 Chernozhukov V, Wüthrich K, Zhu Y. An exact and robust conformal inference method for counterfactual and synthetic controls. *J Am Stat Assoc* 2021; **116**: 1849–64.
